# Picornaviruses and nuclear functions: targeting a cellular compartment distinct from the replication site of a positive-strand RNA virus

**DOI:** 10.3389/fmicb.2015.00594

**Published:** 2015-06-18

**Authors:** Dylan Flather, Bert L. Semler

**Affiliations:** Department of Microbiology and Molecular Genetics, Center for Virus Research, School of Medicine, University of California, IrvineIrvine, CA, USA

**Keywords:** picornavirus, enterovirus, cardiovirus, nucleus, nucleocytoplasmic trafficking, viral RNA replication, IRES

## Abstract

The compartmentalization of DNA replication and gene transcription in the nucleus and protein production in the cytoplasm is a defining feature of eukaryotic cells. The nucleus functions to maintain the integrity of the nuclear genome of the cell and to control gene expression based on intracellular and environmental signals received through the cytoplasm. The spatial separation of the major processes that lead to the expression of protein-coding genes establishes the necessity of a transport network to allow biomolecules to translocate between these two regions of the cell. The nucleocytoplasmic transport network is therefore essential for regulating normal cellular functioning. The *Picornaviridae* virus family is one of many viral families that disrupt the nucleocytoplasmic trafficking of cells to promote viral replication. Picornaviruses contain positive-sense, single-stranded RNA genomes and replicate in the cytoplasm of infected cells. As a result of the limited coding capacity of these viruses, cellular proteins are required by these intracellular parasites for both translation and genomic RNA replication. Being of messenger RNA polarity, a picornavirus genome can immediately be translated upon entering the cell cytoplasm. However, the replication of viral RNA requires the activity of RNA-binding proteins, many of which function in host gene expression, and are consequently localized to the nucleus. As a result, picornaviruses disrupt nucleocytoplasmic trafficking to exploit protein functions normally localized to a different cellular compartment from which they translate their genome to facilitate efficient replication. Furthermore, picornavirus proteins are also known to enter the nucleus of infected cells to limit host-cell transcription and down-regulate innate antiviral responses. The interactions of picornavirus proteins and host-cell nuclei are extensive, required for a productive infection, and are the focus of this review.

## Introduction

### Overview

In this section, we will first provide a brief review of nucleocytoplasmic trafficking in uninfected eukaryotic cells, followed by an outline of the salient features of picornavirus gene expression and replication. Refer to Table 1 for acronyms used in this article.

**Table 1:** **Acronyms used in this article**.

**Acronym**	**Definition**
NPC	Nuclear pore complex
Nup	Nucleoporin
FG	Phenylalanine-glycine-rich
NLS	Nuclear localization signal
NES	Nuclear export signal
NCR	Non-coding region
IRES	Internal ribosome entry site
S-L	Stem-loop
ITAF	IRES trans-acting factors
RNP	Ribonucleoprotein
EMCV	Encephalomyocarditis virus
FMDV	Foot and mouth disease virus
HRV	Human rhinovirus
CVB3	Coxsackievirus B3
EV71	Enterovirus 71
TMEV	Theiler's murine encephalomyelitis virus
HAV	Hepatitis A virus
EGFP	Enhanced green fluorescent protein
PAMP	Pathogen associated molecular pattern
ISG	Interferon-stimulated gene
Pol	RNA polymerase

### The nucleus and nucleocytoplasmic transport

**Figure 1:**
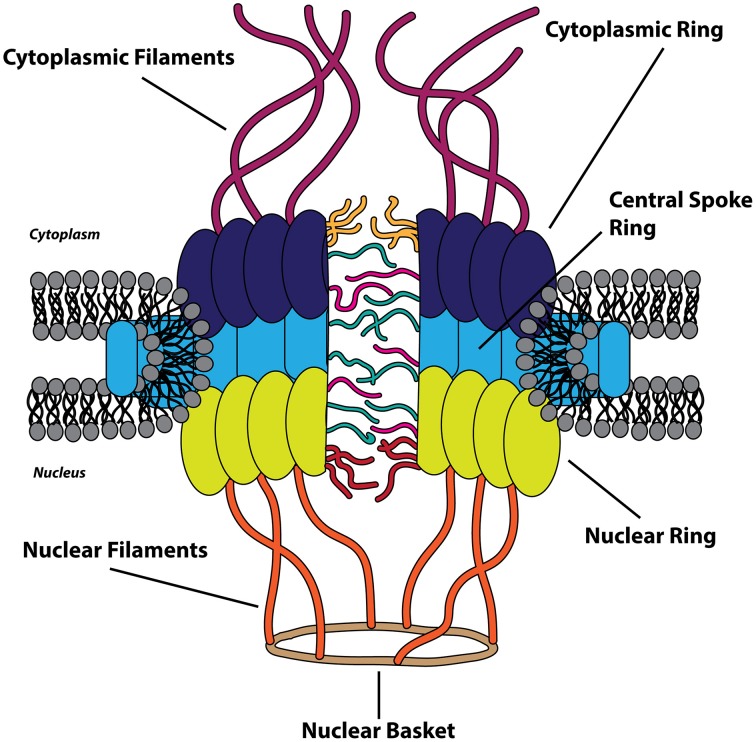
**The nuclear pore complex**. The cytoplasmic (dark blue), central spoke (light blue), and nuclear ring (chartreuse) structures constitute the symmetric portion of the nuclear pore complex (NPC) that surrounds the central channel. The asymmetric portion of the NPC is composed of cytoplasmic filaments (purple) on the cytoplasmic side and the nuclear filaments (orange) and nuclear basket (brown) on the nuclear side of the nuclear envelope. Transmembrane and scaffold nucleoporins are found within the three symmetric ring-like structures of the NPC, and the FG-repeat containing barrier nucleoporins are depicted as filaments within the central channel.

The nucleus is bound by a double membrane of phospholipids termed the nuclear envelope. The inner nuclear membrane is associated with a network of the scleroprotein lamin, comprising the nuclear lamina, and the outer nuclear membrane is an extension of the endoplasmic reticulum (Callan et al., 1949). The nuclear envelope functions as a physical barrier between the cytoplasm and the nucleus and is selectively permeable via nuclear pores, which average in number between 2000 and 5000 per nucleus in vertebrate cells (Grossman et al., 2012). Macromolecules traffic between the nucleus and cytoplasm through these pores that fuse the inner and outer nuclear envelope. Protein complexes known as the nuclear pore complex (NPC) are integrated within the nuclear pores and act as gates that restrict the diffusion of larger biomolecules across the nuclear envelope. With an approximate mass of 125 MDa, the NPC is one of the largest and most complex assemblages of proteins in the eukaryotic cell and is composed of approximately 30 different nucleoporin (Nup) proteins, with ~500–1000 individual Nups comprising a single NPC (Reichelt et al., 1990; Cronshaw et al., 2002; Hoelz et al., 2011). The NPC is a dynamic and modular structure with eight-fold rotational symmetry and can be divided into three recognizable ring-like structures surrounding the central channel of the nuclear pore: the cytoplasmic ring, the central spoke ring, and the nuclear ring (which make up the symmetrical portion of NPC) (Frenkiel-Krispin et al., 2010). Attached to the cytoplasmic ring and nuclear ring are 8 proteinaceous filaments which extend into the cytoplasm and nucleus, respectively, with the nuclear filaments converging to form the nuclear basket (Cautain et al., 2015). These extended structures, together, make up the asymmetric portion of the NPC. Nups are categorized as transmembrane, barrier, or scaffold Nups based upon location within the NPC, amino acid sequence motifs, and structure (Grossman et al., 2012). Transmembrane Nups anchor the NPC to the nuclear envelope pores, barrier Nups facilitate active transport of cargoes, and scaffold Nups link the transmembrane Nups to the barrier Nups, providing the structural framework of the NPC (Figure 1).

Barrier Nups contain repeated phenylalanine-glycine-rich (FG) sequences that form intrinsically disordered motifs and act as the major impediment to free diffusion through the main channel of the NPC (Cautain et al., 2015). Concomitantly, these FG-Nups provide the only route for active transport of cargo biomolecules between the cytoplasm and nucleus by providing binding sites for nuclear transport receptors, within the NPC, through multiple low-affinity interactions (Ben-Efraim and Gerace, 2001; Ribbeck and Görlich, 2001). The translocation of complexes through the NPC is energy-independent as GTP hydrolysis is required only as a final step in the transport process (Schwoebel et al., 1998). The efficiency of nucleocytoplasmic transport is staggering: a single NPC has been proposed to be capable of transporting a 100 kDa protein at an average rate of 800 translocation events per second (Ribbeck and Görlich, 2001; Fried and Kutay, 2003).

**Figure 2:**
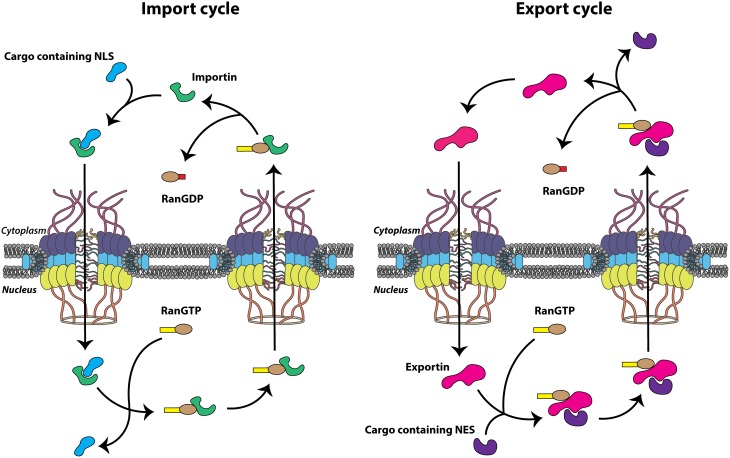
**Nuclear transport cycles**. Nuclear import and export cycles function in complementary fashion to recycle nuclear transport receptors, importins and exportins (green and pink, respectively), through the nuclear pore complex (NPC). Transport of biomolecules (cargo) containing a nuclear localization signal (NLS) (light blue) or nuclear export signal (NES) (purple) through the NPC itself is energy independent but the movement of nuclear transport receptors is dependent upon the hydrolysis of GTP.

Small molecules including ions, metabolites, and proteins less than ~40 kDa are able to translocate between the cytoplasm and nucleus via passive diffusion, perhaps through channels peripheral to the major channel of the NPC (Hinshaw et al., 1992). In addition to allowing this energy-independent diffusion, the NPC simultaneously facilitates the selective, energy-dependent nucleocytoplasmic trafficking of large cellular molecules. This is generally accomplished via specific amino acid sequences present on cargo proteins known as nuclear localization signals (NLSs) or nuclear export signals (NESs), depending on the directionality of transport. These signal sequences are recognized by the soluble nuclear transport receptors that bind cargo proteins and actively transport these molecules through the NPC. Many nuclear transport receptors belong to the karyopherin protein family, known as importins or exportins, and bind specific cargo proteins directly or through adaptor molecules to shuttle proteins from one side of the nuclear envelope to the other. The energy required for this process is provided by GTP hydrolysis carried out by the GTPase Ran, and the concentration gradient of Ran bound to GTP (Ran-GTP) imparts the directionality needed for the proper segregation of nuclear and cytoplasmic functions. Ran-GTP is abundant in the nucleus due to the presence of chromatin-bound Ran-guanine nucleotide exchange factor (Ran-GEF). Conversely, Ran-GDP is more abundant on the cytoplasmic side of the nuclear envelope as a result of the cytoplasmic filament-bound Ran-GTP-activating protein (Ran-GAP), which increases the GTPase activity of Ran, rapidly hydrolyzing bound GTP to GDP (Grossman et al., 2012). Accordingly, the Ran-GTP gradient provides directionality to nucleocytoplasmic transport because importins and exportins utilize the Ran-GTP gradient in a complementary fashion. Nuclear import complexes (importin(s)/cargo) associate at low Ran-GTP concentrations in the cytoplasm, traverse the NPC through transient association-dissociation between importin and FG-Nups. The cargo is then released by the interaction between the import complex and Ran-GTP in the nucleus (Rexach and Blobel, 1995; Görlich et al., 1996). Conversely, trimeric nuclear export complexes (exportin/cargo/Ran-GTP) associate at high Ran-GTP concentrations in the nucleus, traverse the NPC, and dissociate upon interconversion of Ran-GTP to Ran-GDP in the cytoplasm. Both importins and exportins bind Ran-GTP directly and utilize the metabolic energy provided by the Ran-GTPase system to relate directionality to transport (Figure 2). Nucleocytoplasmic transportation is a highly regulated and effective process necessary for cellular homeostasis and, correspondingly, is the target of perturbation by many viral pathogens, including the picornaviruses.

### Picornaviruses

**Figure 3:**
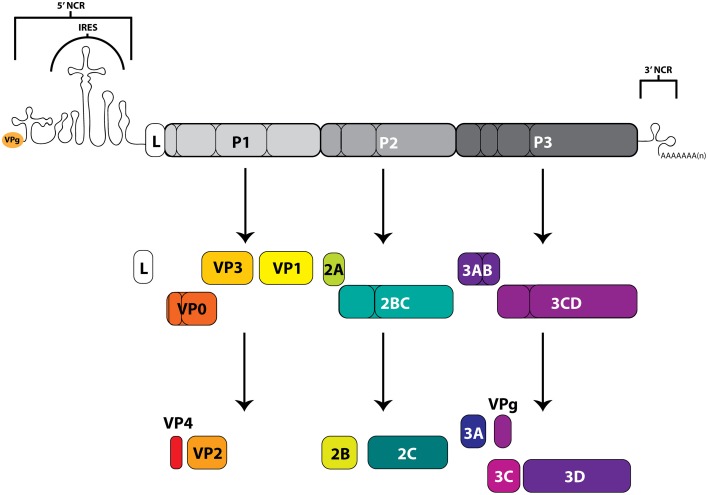
**Picornavirus genome map and polyprotein cleavage cascade**. The positive-sense RNA genomes of picornaviruses contain an internal ribosome entry site (IRES), within a 5′-noncoding region (5′NCR), which drives the cap-independent translation of the downstream open reading frame. Viral gene products include functional precursors (some of which are depicted here) that are further processed by viral proteinases to produce mature viral proteins. The precursor protein 3AB associates with membranes and stimulates the function of 3CD and the RNA-dependent RNA polymerase 3D. The proteolytically active 3CD precursor functions in VPg uridylylation and viral RNA replication through the formation of the ternary complex on the 5′-terminus of viral genomic RNA molecules. Protein 3C functions in viral protein maturation through its proteinase activity. VPg (3B) acts as a protein primer for initiation of viral RNA synthesis. The 2A protein of enteroviruses has proteinase activity, but the 2A of cardioviruses does not. Proteins that form the viral capsid are encoded in the P1 region. Leader protein (L) is not encoded by all picornaviruses, and in some genera (including aphthoviruses), L has proteinase activity. The 3′-terminus of the genome contains a 3′-noncoding region as well as a genetically encoded poly(A) tract.

The picornaviruses are a large group (26 genera currently recognized) of non-enveloped, small (~30 nm in diameter) viruses containing a positive-polarity, single-stranded RNA genome of ~7–9 kb in length with a viral protein (VPg) covalently attached to the 5′-terminus of the genome. These RNA molecules contain both 5′ and 3′ untranslated regions that function, in association with viral and host cell proteins, to facilitate both translation of the single open reading frame flanked by these regions, as well as RNA replication for genomic amplification. The long (~600–1500 nucleotide, including up to a ~500 nucleotide poly(C) tract for some aphthoviruses), highly structured, 5′-noncoding region (NCR) contains an internal ribosome entry site (IRES) that directs the cap-independent translation of a large polyprotein from the viral genome (Racaniello, 2013; Martínez-Salas et al., 2015). This precursor polyprotein is co- and post-translationally processed by viral proteinase 3CD (as well as enteroviral 2A and leader proteinase L for aphthoviruses and erboviruses) to generate intermediate and mature viral proteins with distinct functions. In addition to the highly structured IRES region, many picornaviruses also contain stem-loop (S-L) structures important for protein interactions that promote genome replication. On the other terminus of the viral genome, the shorter (~50–350 nucleotide) 3′-NCR contains structured regions involved in viral RNA synthesis (though non-essential for infectivity), as well as an essential poly(A) tract (Racaniello, 2013). The RNA-dependent RNA polymerase, 3D, functions to replicate the viral genome through a negative-stranded intermediate, and is encoded within the P3 region of the polyprotein (Figure 3).

The infectious cycles of picornaviruses are initiated following viral attachment to specific cellular receptors. The RNA genome is then released from the virion capsid and enters the cytoplasm of the infected cell. Once in the cytoplasm, the picornavirus RNA molecule is used as a template for IRES-driven viral protein production. An incompletely defined set of events that likely involve local concentrations of viral proteins and cleavage of specific host factors allows the same RNA template used for translation to be cleared of ribosomes and utilized for the production of a complementary, intermediate negative-sense (anti-genomic) RNA molecule, producing a double-stranded RNA structure called the replicative form. This opposite polarity molecule is, in turn, used for the production of positive-sense RNA molecules, generating a multiple-stranded RNA complex composed of negative-sense RNA templating multiple genomic RNA molecules simultaneously, called the replicative intermediate. These nascently produced molecules are then recycled back through the translation/replication process or packaged into progeny virions. Importantly, proteins predominantly localized to the nucleus in uninfected cells are utilized by these cytoplasmic viruses from the very primary steps of the replication process.

## Nuclear-resident proteins are hijacked for picornavirus translation

The positive-sense RNA genome of a picornavirus is competent for immediate IRES-driven translation upon uncoating. In addition to picornavirus RNAs, it has been suggested that up to 10% of cellular mRNAs contain an IRES element (Spriggs et al., 2005). Somewhat counterintuitively, many of the proteins that mediate IRES-dependent translation of cellular and viral mRNAs, known as IRES trans-acting factors or ITAFs, are compartmentalized in the host cell nucleus or shuttle between the nucleus and the cytoplasm (Semler and Waterman, 2008). It is currently unclear whether ITAFs associate with cellular IRES elements during the biogenesis of mRNA transcripts in the nucleus and are subsequently transported to the cytoplasm as ribonucleoprotein (RNP) complexes, or whether these ITAFs are redistributed to the cytoplasm, where IRES-containing mRNAs are already present, in response to signals stimulating IRES-driven translation. Regardless of the mechanism in which ITAFs associate with cellular IRES elements, the ITAFs utilized by picornaviruses are available at sufficient concentration in the cytoplasm upon infection, at least for the initial rounds of viral protein production. Importantly, picornaviruses subvert the host protein synthesis machinery by cleavage of canonical translation factors, inhibiting cellular translation and releasing ribosomes and associated proteins from their roles in cap-dependent translation. For the purpose of discussion, nuclear-resident proteins will be defined as those that are normally more concentrated in the nucleus than the cytoplasm, because all cellular proteins can be found to some extent in the cytoplasm during biogenesis and many shuttle between the nucleus and cytoplasm to perform their functions. Importantly, these nuclear-resident/shuttling proteins often have RNA-binding capabilities and control many features of RNA biology and gene expression including: splicing, mRNA transport out of the nucleus, and mRNA stability. Furthermore, mammalian cells encode nearly 1000 RNA-binding proteins (although not all of these are nuclear-resident) and as a result, the viral mRNAs of picornaviruses employ the functions of several of these RNA-binding and RNA-chaperone proteins to facilitate translation (Castello et al., 2012).

While all picornaviruses contain IRES elements within the 5′-NCR of their genomes to facilitate ribosome association, these elements are categorized into four separate types, I–IV, for the 12 best studied picornavirus genera, depending upon primary RNA sequence, secondary RNA structure, location of translation initiation codon, and phylogeny. Type I structures are found in the genomes of enteroviruses; Type II in aphthoviruses, cardioviruses, erboviruses, kobuviruses, and parechoviruses; Type III in hepatoviruses; and Type IV in avihepatoviruses, sapeloviruses, senecaviruses, teschoviruses, and tremoviruses (Palmenberg et al., 2010; Martínez-Salas et al., 2015). The categorization of picornavirus IRES types is somewhat arbitrary and flexible, especially as work related to cap-independent translation from these viruses continues. Recently, it has been proposed that the Kobuvirus genus contains a distinct, fifth type of IRES, but there will be no further discussion within this review, as no nuclear-resident ITAFs have been reported for this IRES type (Sweeney et al., 2012). There is little sequence homology across the four IRES types and, as a result, picornaviruses harboring different IRES structures likely utilize slightly different cohorts of ITAFs and in different ratios. However, there is at least some overlap in the identity of those nuclear proteins that are used as ITAFs for general picornavirus translation. ITAFs often have functions in the uninfected cell related to transcriptional regulation, splicing, and RNA transport and stability (Martínez-Salas et al., 2015).

### Polypyrimidine tract-binding protein 1 (PTBP1)

Type I and Type II IRESs have been the most extensively studied of the picornavirus IRES elements and correspondingly, have been shown to associate with the greatest number of nuclear-resident proteins compared to the other IRES Types. Polypyrimidine tract-binding protein 1 (PTBP1 also known as hnRNP I) was the first host protein shown to interact with, and promote translation from, the IRES regions of encephalomyocarditis virus (EMCV), foot and mouth disease virus (FMDV), poliovirus, and human rhinovirus (HRV) 2 (Jang and Wimmer, 1990; Luz and Beck, 1991; Hellen et al., 1993; Kaminski et al., 1995; Niepmann, 1996; Hunt and Jackson, 1999). PTBP1 contains four RNA recognition motifs distributed across a flexible structure, functions in the regulation of pre-mRNA splicing and transport, and has been shown to be predominantly localized to the nucleus while also shuttling to the cytoplasm (Ghetti et al., 1992; Oh et al., 1998; Sawicka et al., 2008). This protein is hypothesized to promote translation initiation on Type I IRESs by modulating an interaction between domain V of these structures and the C-terminal portion of translation initiation factor eIF4G, which is cleaved during infection with enteroviruses but retains some RNA binding capability, with the C-terminal fragment utilized for cap-independent translation (Buckley and Ehrenfeld, 1987; Ohlmann et al., 1995, 1996; Kafasla et al., 2010). Coxsackievirus B3 (CVB3), which contains a Type I IRES element, also utilizes PTBP1 as an ITAF, and because it has been shown to bind both the 5′- and 3′-NCRs of CVB3 RNA, has been proposed to facilitate circularization of the RNA molecule to promote efficient translation (Verma et al., 2010). Furthermore, EMCV and FMDV Type II IRESs appear to require the binding of two copies of PTBP1, at two distinct regions, for maximal IRES activity, at least *in vitro* (Kolupaeva et al., 1996; Kafasla et al., 2009). PTBP1 likely acts as an RNA chaperone, stabilizing viral IRES structures, since poliovirus and EMCV translation is dependent upon the simultaneous interaction of three of the four RNA-binding domains found within this protein, and FMDV requires two of the four RNA-binding domains of PTBP1 for efficient IRES activity (Song et al., 2005; Kafasla et al., 2011). PTBP1 has been demonstrated to be the only ITAF (non-canonical translation factor) that is required for the translation of EMCV transcripts *in vitro* (Pestova et al., 1996).

### Lupus La protein (La)

The Lupus La protein (La, also known as La autoantigen) has also been implicated in having a role in the cap-independent translation of type I and II IRESs. La has been shown to bind a portion of the poliovirus IRES as a dimer and enhance the production of poliovirus proteins (Meerovitch et al., 1989, 1993; Craig et al., 1997). Similarly, La protein stimulates the translation of both CVB3 and EMCV RNA (Kim and Jang, 1999; Ray and Das, 2002). La functions to stabilize nascent RNA produced in cells. It binds the 3′ poly(U) termini of RNA polymerase III transcripts to protect and promote maturation of these RNA molecules, and as a result is generally confined to the nucleus (Stefano, 1984). La has been proposed to mediate an interaction between the 40S ribosomal subunit and the poliovirus IRES *in vivo* (Costa-Mattioli et al., 2004).

### Poly(rC)-binding protein 2 (PCBP2)

Poly(rC)-binding protein 2 (PCBP2) binds single-stranded nucleic acids through three hnRNP K-homologous domains (KH domains) and is involved in the stabilization of several cellular mRNAs (Siomi et al., 1994; Holcik and Liebhaber, 1997). The predominantly nuclear PCBP2 binds to both stem-loop IV (S-L IV) and stem-loop I (S-L I) of the 5′-NCR within the poliovirus and CVB3 genomic RNA (Blyn et al., 1995, 1996; Leffers et al., 1995; Gamarnik and Andino, 1997; Parsley et al., 1997; Zell et al., 2008a,b; Sean et al., 2009). S-L IV is located in the central portion of Type I IRES elements and alterations to the nucleic acid sequence identified as important for PCBP2 association decrease poliovirus translation *in vitro* (Blyn et al., 1995). Moreover, depletion of PCBP2 from cellular extracts results in inefficient poliovirus translation (Blyn et al., 1997). Although PCBP2 is required for translation of poliovirus, coxsackievirus, and HRV, it is not necessary for the translation of the type II IRES-containing RNAs of EMCV and FMDV (Walter et al., 1999). PCBP2 is the only ITAF shown to be required for the translation of poliovirus, enterovirus 71 (EV71), and bovine enterovirus (i.e., Type I IRESs) by *in vitro* reconstitution of translation initiation (Sweeney et al., 2014). However, as with PTBP1 and the EMCV IRES, whether these *in vitro* systems are representative of the conditions encountered within the cellular milieu during infection, or if other, non-essential, ITAFs enhance viral IRES-driven translation, remains to be elucidated.

### Serine/arginine-rich splicing factor 3 (SRSF3)

Type I IRES structures also utilize serine/arginine-rich splicing factor 3 (SRSF3 or SRp20) to promote cap-independent translation. The SR proteins comprise a group of splicing factors with a multitude of functions related to gene expression including: constitutive and alternative splicing, mRNA export and stability, and translation (Graveley, 2000; Huang and Steitz, 2001; Huang et al., 2003; Sanford et al., 2004). As a result of these functions, a subset of SR proteins including SRSF3 are considered shuttling proteins, although they most often accumulate in the cellular nucleus (Cáceres et al., 1998). Depletion of SRSF3 from cells or cellular extracts decreases the protein production from a reporter construct containing the poliovirus IRES (Bedard et al., 2007). In addition, SRSF3 and PCBP2 have been shown to act synergistically to increase the efficiency of IRES-mediated translation *in vitro* and in poliovirus-infected cells, with SRSF3 associating with S-L IV of the IRES via a PCBP2 bridge. Specifically, this enhancement in non-canonical translation is a result of SRSF3 interacting with the KH3 domain of PCBP2 to directly or indirectly recruit ribosomes to the viral RNA. Furthermore, both are found associated with translation initiation complexes in poliovirus-infected cells (Bedard et al., 2007; Fitzgerald and Semler, 2011). CVB3 and HRV 16 also likely utilize SRSF3 to promote translation, as this protein is relocalized in cells expressing the 2A proteinase of these viruses (Fitzgerald et al., 2013).

### Proliferation-associated protein 2G4 (PA2G4)

The SRSF3/PCBP2 cooperative enhancement to poliovirus IRES-driven translation initiation is mirrored by the interaction between PTBP1 and proliferation-associated protein 2G4 (PA2G4 or EBP1) with the FMDV IRES element. A chimeric Theiler's murine encephalomyelitis virus (TMEV, which also utilizes a Type II IRES) containing the FMDV IRES element in place of the TMEV IRES was unable to replicate in mouse neurons, suggesting the absence of a necessary ITAF for minimal FMDV translation. This ITAF was identified as PA2G4 (“ITAF 45”) in assaying for the formation of 48S initiation complexes through biochemical reconstitution and was shown to bind directly to viral RNA corresponding to the FMDV IRES through UV cross-linking (Pilipenko et al., 2000). PA2G4 and PTBP1 bind to distinct sites within the FMDV IRES, causing localized structural changes within these regions, thereby enhancing binding of the eIF4G/4A complex to the IRES structure. It has been proposed that unlike in the case of the TMEV IRES, PTBP1 alone is unable to promote the RNA structural modifications to the FMDV IRES necessary for ribosome association and translation initiation. This is likely due to differences in the nucleotide sequence within these Type II IRES structures and associated differences in the way in which PTBP1 binds and arranges these regions, forcing FMDV to rely on PA2G4 to shape a functionally competent IRES structure (Pilipenko et al., 2000). PA2G4 was also shown to interact with TMEV and EMCV RNA, so this protein likely binds RNA non-specifically. However, EMCV IRES-driven translation has been shown to be unaffected by the presence of PA2G4, and PA2G4 does not interact with PTBP1, corroborating the fact that translation initiation from the EMCV IRES is independent of PA2G4 (Monie et al., 2007). Interestingly, despite the fact that FMDV and EMCV have different ITAF requirements, experiments comparing the sites of hydroxyl radical cleavage within the IRES structures from the eIF4G hub demonstrated that when these Type II IRESs interact with their cognate ITAFs, similar structural conformations are adopted (Yu et al., 2011). This suggests that although these IRES sequences can vary by ~50%, their shared requirement for PTBP1 seems to lie in the fact that it acts as versatile adaptor protein, whether alone or in combination with other ITAFs, in translation initiation form Type II IRESs.

### Minimal vs. stimulatory ITAFs

It is important to note that while there has been rather extensive study of the Type I and Type II IRESs and their associated ITAFs, there is some variability in the reported ITAF requirements across particular viral species. For the Type I IRES elements tested (poliovirus, HRV 2, and CVB3), PTBP1 has been shown to be both required or unnecessary (Hunt and Jackson, 1999; Verma et al., 2010; Sweeney et al., 2014). Similarly, for viruses containing Type II IRES elements, there is discrepancy in the obligatory ITAFs reported. PTBP1 is required for FMDV translation, but the requirement for this ITAF in EMCV translation appears conditional upon the reporter and IRES variant utilized in experiments (Kaminski and Jackson, 1998). Additionally, translation from the TMEV IRES has been shown to be independent of, as well as strongly dependent on, PTBP1 (Kaminski et al., 1995; Pilipenko et al., 2001). As mentioned previously, the inconsistencies in reported ITAFs is likely a result of the assay used (*in vitro* compared to experiments in cells) as well as the use of different strains of virus/sequences of reporter constructs. These apparently different results likely point to the fact that there are some minimal ITAFs required for Type I and Type II IRES elements but a multitude of ITAFs that play some stimulatory or translation-enhancing role depending on the specific context of an infection. For example, it is possible that different ITAFs are utilized by viral IRES elements depending on the cell type infected (i.e., cell-type-specific ITAFs), as the availability of particular proteins that function in this regard likely dictate whether viral protein production and growth are supported, and to what extent (Chang et al., 1993; Wimmer et al., 1993). Cell-type-specific IRES function is exemplified by the fact that viral RNAs that initiate translation from the HRV 2 IRES, but not the poliovirus IRES, are excluded from neuronal cell polysomes but not from those of glioma cells. This is thought to be the result of a specific protein heterodimer that inhibits HRV 2 IRES-driven translation in neuronal but not glioma cells, as discussed below (Merrill and Gromeier, 2006).

### Other ITAFs of type I IRESs

Other nuclear-resident proteins that function as ITAFs of Type I IRESs have also been proposed, but less completely characterized. Nucleolin, which relocalizes to the cytoplasm of poliovirus-infected cells, has been shown to stimulate translation from constructs containing poliovirus and rhinovirus IRES structures *in vitro* and in cells, and the amino-terminal domain of this protein is important for this activity (Waggoner and Sarnow, 1998; Izumi et al., 2001). hnRNP A1 shuttles between the nucleus and the cytoplasm, functions in both pre-mRNA splicing and nuclear export of mRNA molecules, and has been shown to interact with the EV71 IRES via electrophoretic mobility shift assay. Furthermore, when hnRNP A1 and hnRNP A2 are knocked down in cells, there is a decrease in translation of a reporter gene containing the EV71 IRES sequence, and there is an overall reduction in viral replication in these cells (Lin et al., 2009b). Similar results have also been observed with the EV71 IRES and far upstream element-binding protein 1 (FBP1) (Huang et al., 2011). It has also been reported that the Type II IRES-containing FMDV may utilize the nuclear KH domain-containing, RNA-binding, signal transduction-associated protein 1 (Sam68) to promote IRES-dependent protein production following L-protein dependent cleavage and subsequent cytoplasmic localization (Lawrence et al., 2012).

### Nuclear-resident proteins that inhibit picornavirus translation

In contrast to the discussion thus far, there are also examples of nuclear-resident proteins that act to inhibit enterovirus translation. Double-stranded RNA binding protein 76 (DRBP76, also known as interleukin enhancer-binding factor 3) heterodimerizes with nuclear factor of activated T cells, 45 kDa (NF45, also known as interleukin enhancer-binding factor 2), and inhibits translation initiation from the HRV 2 IRES in neuronal cells. The recombinant oncolytic poliovirus PVS-RIPO exploits the incorporated HRV 2 IRES to permit attenuated neurovirulence in the treatment of malignant glioma, possibly due to the presence of DRBP76:NF45 heterodimers in the cytoplasm of neuronal cells (Merrill et al., 2006; Merrill and Gromeier, 2006). Similarly, far-upstream element-binding protein 2 (FBP2 or KSRP), which is involved in splicing and mRNA trafficking, inhibits EV71 IRES-driven translation and is cleaved during infection (Lin et al., 2009a; Chen et al., 2013). Cytoplasmic proteins that function as ITAFs for picornavirus RNA translation have also been reported, as well as canonical elongation factors, but these are outside the scope of this review (for a recent review of picornavirus translation including the role of cytoplasmic proteins, see Martínez-Salas et al., 2015).

### ITAFs of type III and type IV IRESs

There has been comparatively little study of type III and IV IRES elements, but several of the same nuclear-resident proteins involved in IRES-driven translation of type I and type II IRES structures have been implicated for type III structures as well. The type III IRES structure found in the genome of hepatitis A virus (HAV) interacts with, is stabilized by, and is stimulated by PTBP1 (Chang et al., 1993; Gosert et al., 2000a). Similarly, HAV IRES activity is increased in the presence of PCBP2. However, although this protein interacts with the 5′-NCR of the HAV genome, it does not bind to regions that correspond to the IRES structure (Graff et al., 1998). Interestingly, in contrast to its role in the functions of type I and type II IRESs, La suppresses translation from the HAV IRES (Cordes et al., 2008). Even less is known about the ITAF requirements of picornavirus type IV IRES elements, but as these IRES elements are very similar to those found in some Flaviviruses, it is expected that at least some of the same ITAFS utilized by hepatitis C virus, for example, also enhance the translation of sapelovirus, senecaviruses, teschoviruses, and tremoviruses.

### Other nuclear-resident proteins that interact with viral RNA molecules

Large-scale proteomic studies have identified a multitude of RNA-binding proteins that interact with poliovirus RNA isolated from infected cells as well as FMDV RNA *in vitro*, but the specific role of each of these identified proteins remains to be elucidated (Pacheco et al., 2008; Lenarcic et al., 2013). One protein identified as interacting with the FMDV IRES element through large-scale proteomic studies is Gem-associated protein 5 (Gemin5), which was shown to inhibit FMDV translation, likely by competitively inhibiting PTBP1 binding (Piñeiro et al., 2013). Although those proteins that act as ITAFs to mediate the translation of picornavirus RNA templates warrant further investigation, one commonality among the proteins discussed above is that they are all RNA-binding proteins with the ability to form multimers. This suggests that they are able to interact with the viral IRESs in multiple locations and perhaps stabilize the structures of their associated IRESs to promote recognition by the translation machinery (Jackson et al., 1995; Kafasla et al., 2009) (Table 2).

**Table 2:** **Nuclear-resident proteins involved in picornavirus translation**.

**Nuclear-resident protein**	**Picornavirus IRES type**	**Effect**	**References**
Polypyrimidine tract-binding protein 1 (PTBP1)	I, II, III	Promotes translation through stabilization of IRES structure	Jang and Wimmer, 1990; Luz and Beck, 1991; Chang et al., 1993; Hellen et al., 1993; Kaminski et al., 1995; Kolupaeva et al., 1996; Niepmann, 1996; Pestova et al., 1996; Hunt and Jackson, 1999; Gosert et al., 2000a; Song et al., 2005; Kafasla et al., 2009, 2010; Verma et al., 2010; Kafasla et al., 2011
Lupus La protein (La)	I, II, III	Stimulates translation from Type I and Type II IRESs; suppresses translation from Type III IRES structures	Meerovitch et al., 1989, 1993; Craig et al., 1997; Kim and Jang, 1999; Ray and Das, 2002; Costa-Mattioli et al., 2004; Cordes et al., 2008
Poly(rC)-binding protein 2 (PCBP2)	I, III	Stimulates translation from Type I and Type III IRESs but does not bind Type III IRES element	Blyn et al., 1995, 1996; Gamarnik and Andino, 1997; Parsley et al., 1997; Graff et al., 1998; Zell et al., 2008a,b; Sean et al., 2009; Sweeney et al., 2014
Serine/arginine-rich splicing factor 3 (SRSF3 or SRp20)	I	Acts synergistically with PCBP2 to increase efficiency of poliovirus translation	Bedard et al., 2007; Fitzgerald and Semler, 2011
Proliferation-associated protein 2G4 (PA2G4 or EBP1)	II	Necessary for FMDV translation but not required for TMEV or EMCV translation	Pilipenko et al., 2000; Monie et al., 2007
Nucleolin	I	Stimulates translation	Waggoner and Sarnow, 1998; Izumi et al., 2001
Heterogeneous nuclear ribonucleoprotein A1 (hnRNP A1)	I	Interacts with EV71 IRES, depletion results in reduced reporter gene translation	Lin et al., 2009b
Far upstream element-binding protein 1 (FBP1)	I	Interacts with EV71 IRES	Huang et al., 2011
KH domain-containing, RNA-binding, signal transduction-associated protein 1 (Sam68)	II	Promotes FMDV translation	Lawrence et al., 2012
Double-stranded RNA binding protein 76 (DRBP76)	I	Heterodimerizes with nuclear factor of activated T-cells, 45 KDa (NF45) and inhibits HRV 2 translation	Merrill et al., 2006; Merrill and Gromeier, 2006
Far-upstream element-binding protein 2 (FBP2)	I	Inhibits EV71 IRES-driven translation	Lin et al., 2009a; Chen et al., 2013
AU-rich binding factor 1 (AUF1 or hnRNP D0)	I	Binds IRES element and inhibits translation	Cathcart et al., 2013; Wong et al., 2013; Lin et al., 2014
Gem-associated protein 5 (Gemin5)	II	Likely inhibits FMDV translation through competitive inhibition of PTBP1 binding	Piñeiro et al., 2013

### Nuclear-resident proteins function in template-usage switching

In addition to the roles that nuclear RNA binding proteins play in viral translation, they may also govern the template usage switch that occurs following the production of picornavirus proteins to transition to viral RNA replication. The same genomic template is used for both translation and RNA replication; however this RNA but cannot be traversed simultaneously by ribosomes and the viral polymerase, which travel in opposite directions on the template. Thus, the RNA must be “reset” prior to RNA replication (Barton et al., 1999). The regulation of this switch is dependent, in part, upon the sufficient production of viral proteins, specifically proteinases, which subsequently target the ITAFs that initially promoted the translation of the proteinases themselves. As mentioned previously, PCBP2 binds to both S-L I and S-L IV of poliovirus genomic RNA, but with much greater affinity to S-L IV in isolation (Gamarnik and Andino, 2000). However, upon cleavage by the viral 3CD/3C proteinase, the cleaved PCBP2 is unable to stimulate IRES-driven translation. 3CD/3C liberates the C-terminal KH3 domain, leaving the N-terminal portion of the protein unable to form a complex with S-L IV and therefore unable to aid in ribosome recruitment (Perera et al., 2007). The N-terminal portion of PCBP2 is, however, still capable of interacting with S-L I RNA structures, an interaction that is enhanced when the viral proteinase precursor 3CD is present on this 5′-terminal RNA structure (Gamarnik and Andino, 1998; Perera et al., 2007). The resulting ternary complex formed between S-L I RNA, PCBP2, and 3CD promotes viral RNA synthesis (Parsley et al., 1997; Gamarnik and Andino, 2000). Although the interaction between PCBP2 and S-L I may aid in the stimulation of viral protein synthesis early during infection, it is the presence of 3CD and subsequent interactions with PCBP2 that allows the viral RNA replication process to proceed (Kempf and Barton, 2008). Adding further regulation to the template usage switch orchestrated by poliovirus is the fact that PTBP1, another ITAF, is cleaved by 3CD/3C leading to inhibition of poliovirus translation (Back et al., 2002). Similarly, cleavage of the nuclear shuttling polyadenylate-binding protein 1 (PABP1), which functions in regulation of mRNA metabolism and is closely associated with RNA and mRNP complexes, has been proposed to be involved in the inhibition of poliovirus translation because expression of PABP1 resistant to poliovirus 3C mediated cleavage during infection increases viral protein synthesis from non-replicating reporter RNAs and reduces viral RNA accumulation, compared to wild type PABP1 expression (Afonina et al., 1998; Bonderoff et al., 2008). HAV likely utilizes a similar method to proceed from translation to RNA replication, since the 3C proteinase of this virus has been shown to cleave PCBP2 and PTBP1, resulting in reduced protein-RNA affinity and suppression of translation (Zhang et al., 2007; Kanda et al., 2010). Increased viral protein accumulation and alterations to nuclear alterations to nuclear RNA-binding proteins is the mechanism by which poliovirus and HAV RNA replication is able to proceed, but whether this mechanism to induce a template usage switch is broadly applicable to other picornaviruses is not clear. Indeed, a recent report demonstrated that three HRV serotypes do not induce the cleavage of PCBP2 or PTBP1 during infection of a human lung cell line, suggesting alternative mechanisms must be used under theses experimental conditions (Chase and Semler, 2014).

### A nuclear-resident protein removes VPg from viral genomic RNA molecules

Finally, in addition to the nuclear-resident proteins utilized by picornaviruses to promote IRES-mediated protein production, another nuclear-resident protein functions in RNA processing of poliovirus RNA. VPg, the viral protein covalently attached to the 5′-terminus of the viral RNA, is cleaved from viral RNAs found on polysomes (Ambros et al., 1978). This cleavage is performed by 5′-tyrosyl–DNA phosphodiesterase-2 (TDP2), which normally functions to remove covalent adducts from DNA via hydrolysis of 5′-phosphodiester bond (Virgen-Slane et al., 2012). VPg serves as the protein primer for viral RNA synthesis and is present on encapsidated RNA molecules, but the functional role of TDP2 in viral infection and its VPg cleavage capability is not completely clear. Independent of understanding the precise role of TDP2 during infection, this protein provides an additional example of nuclear-resident proteins being used in diverse ways, even during the very initial steps in the infectious cycles of picornaviruses.

During the initial rounds of IRES-mediated translation, picornaviruses co-opt nuclear shuttling proteins that are encountered in the cytoplasm of an infected cell. As genome amplification proceeds and further rounds of translation are initiated however, increasing amounts of nuclear-resident proteins are required in the cytoplasm of cells to facilitate a productive infectious cycle. To provide these critical nuclear factors to the sites of viral replication, alterations to nucleocytoplasmic trafficking and the normal compartmentalization of cellular proteins occurs, resulting in a large cytoplasmic stock of nuclear factors. How this is achieved is the focus of a subsequent section of this review. So, despite the fact that the early rounds of viral translation and genomic replication can proceed utilizing the limited supply of nuclear-resident proteins already in the cytoplasm, successive rounds require the selective loss of cellular protein compartmentalization allowing cellular factors to be available for viral replication processes. It is clear that nuclear resident proteins play a critical role in the regulation of picornavirus translation, despite that fact that these positive-sense RNA viruses complete their replication cycle in the cytoplasm of the infected cell.

## Nuclear-resident proteins utilized in the process of picornavirus RNA replication

Although picornaviruses encode their own RNA-dependent RNA polymerase (3D), they utilize host cell factors in order to augment the function of this replicase enzyme. As with IRES-dependent translation, most of the factors utilized in the process of RNA synthesis are nuclear-resident proteins with RNA-binding functions that can be used by the virus to facilitate the replication of viral RNA molecules. These host proteins act in the context of RNP complexes they form with picornavirus RNAs to impart replication specificity to the polymerase, as 3D is able to replicate RNA non-specifically when provided with a primed template *in vitro* (Tuschall et al., 1982). During an infection, however, 3D solely replicates picornavirus RNA despite the large excess of cellular mRNA present. To make use of picornavirus RNA templates exclusively, complexes of picornavirus RNA, host nuclear-resident proteins, and viral proteins are thought to act as recognition elements that enable template recognition by 3D and initiate RNA replication. Particularly critical in the regulation of 3D appears to be 5′-3′ intramolecular interactions that yield circularized templates. This section will focus on those nuclear-resident proteins that are known to promote either the production of intermediate negative-sense RNA molecules or the amplification of positive-sense RNA molecules from this template.

As discussed previously, a template usage switch from translation to RNA replication occurs as viral proteins accumulate and, in some cases, proteinases cleave host cell factors functioning as ITAFs. Prior to this transition, it has been proposed that optimal translation of poliovirus RNA requires the circularization of the RNA molecule, likely allowing ribosomes to be efficiently reloaded on the template (Ogram et al., 2010). This finding supports the fact that picornavirus RNA molecules that are to be replicated must first be translated (Novak and Kirkegaard, 1994). Host proteins present on the viral RNA that allow for the initial circularization of the translation-competent template could enhance the efficiency of the circularization of the replication-competent template. This coupling between translation and RNA replication has been suggested to be promoted by at least two common proteins: PABP1 and PCBP2.

### The possible role of nuclear-resident proteins in promoting enterovirus genomic RNA circularization and negative-sense RNA production

**Figure 4:**
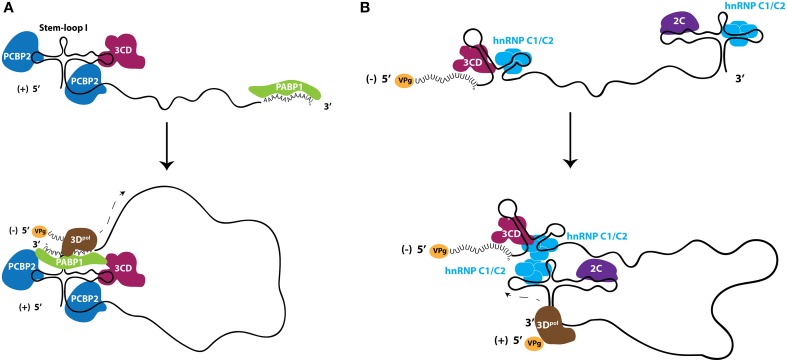
**Ribonucleoproteins (RNPs) comprised of nuclear-resident proteins facilitate enterovirus RNA replication. (A)** Nuclear-resident proteins PCBP2 (dark blue) and PABP1 (green) act in conjunction with viral protein 3CD (fuchsia) to circularize genomic RNA for use as templates to produce negative-sense RNA intermediates. **(B)** Nuclear protein hnRNP C1/C2 (light blue) interacts with both termini of negative-sense RNA molecules and is hypothesized to circularize the negative-sense template to promote genomic RNA production. Although likely in the form of double-stranded RNA, the negative-sense RNA is shown here as single stranded for clarity. Viral protein 2C (purple) interacts with the 5′-terminus of negative-sense RNA, although the direct function of this protein in viral RNA replication is unclear. The viral RNA-dependent RNA polymerase 3D^pol^ (brown) is recruited to these circularized templates and initiates viral RNA synthesis. VPg (yellow), the viral protein that primes RNA synthesis, is found on RNA molecules that have not been translated.

The 5′ non-coding regions of picornavirus genomes contain RNA structural elements that are required for the replication of these genomes by acting as scaffolds for protein interactions (Andino et al., 1990; Barton et al., 2001; Nateri et al., 2002; Nagashima et al., 2008). Electrophoretic mobility shift assays incorporating recombinant proteins and subgenomic portions of poliovirus RNA molecules have been instrumental in identifying the components of RNP elements *in vitro*, that may be important for the process of enterovirus RNA replication. The 5′ terminal structure of the poliovirus genome, known as S-L I or cloverleaf, has been shown to be critical for the formation of RNP complexes that function in the initiation of RNA synthesis (Andino et al., 1993). One of the proteins involved in this RNP formation is the nuclear-resident PCBP2, which binds to the S-L I structure with increased affinity when the viral polymerase precursor, 3CD, is also present near the 5′-terminus of the RNA, forming a ternary complex (Gamarnik and Andino, 1997, 2000; Parsley et al., 1997). On the opposite terminus of the genome, PABP1 associates with the genetically encoded poly(A) tract of the poliovirus genome. Through co-immunoprecipitation using antibodies directed against PABP1, it has been demonstrated that 3CD and PABP1 directly interact in poliovirus-infected cells. As a result, it has been proposed that PABP1 acts as a bridge to link both ends of the viral genome because it is able to simultaneously interact with the 3′-terminus of poliovirus genomic RNA as well as both 3CD and PCBP2, which are present on the 5′-terminus of the same RNA molecule (Herold and Andino, 2001). More recently it has been shown that PCBP2 binds to both the S-L I structure and to a C-rich spacer region that is found between S-L I and the IRES element (Toyoda et al., 2007). Based on similar interactions between PCBP2 and CVB3 RNA, it is likely that PCBP2 modulates the RNA replication of this closely related virus, and the poly(C) binding protein hnRNP K may be exploited in place of PCBP2 by EV71 (Lin et al., 2008; Zell et al., 2008a,b) (Figure 4A).

The nuclear Sam68 which is a putative regulator of mRNA stability and mRNA nuclear export, has been identified as interacting with poliovirus 3D through yeast two-hybrid assays; however, the role this protein plays in the viral RNA replication cycle is not clear (McBride et al., 1996; Coyle et al., 2003). Non-POU domain-containing octamer-binding protein (NONO), identified as interacting with poliovirus RNA through thiouracil cross-linking mass spectrometry, also impacts the generation of positive-sense RNA during infection (Lenarcic et al., 2013). Another nuclear-resident protein with a proposed role in picornavirus replication is RNA Helicase A (RHA), which binds the 5′-NCR of FMDV genomic RNA (Lawrence and Rieder, 2009).

### A nuclear-resident protein promotes enterovirus genomic RNA production

The production of negative-sense viral RNA from genomic templates results in the formation of a double-stranded RNA molecule called the replicative form. The replicative form structure contains the template for the production of genomic RNA, and therefore the duplexed RNA strands within this structure must be separated at the 3′-terminus of the negative-strand RNA to allow for 3D association and the initiation of RNA synthesis. A single host-cell protein, heterogeneous nuclear ribonucleoprotein C1/C2 (hnRNP C1/C2), has been demonstrated to promote the amplification of positive-strand poliovirus RNA from the negative-strand template and, as with the majority of other host proteins involved in the infectious cycles of picornaviruses, is a nuclear-resident protein. hnRNP C1 and C2 are produced by alternative splicing, with the C2 isoform containing 13 additional amino acids (Koloteva-Levine et al., 2002). Together, these hnRNP C1 and C2 proteins form a heterotetramer containing three copies of C1 and a single copy of C2 that bind pre-mRNA, regulate splicing, and nucleate the formation of 40S hnRNP particles (Barnett et al., 1989; Huang et al., 1994). Each C protein contains an RNA recognition motif, an oligomerization domain, a nuclear localization signal, and a nuclear retention signal (Görlach et al., 1992; McAfee et al., 1996; Nakielny and Dreyfuss, 1996; Wan et al., 2001). In contrast to many other hnRNP proteins, hnRNP C1/C2 appears to be restricted to the nucleus and does not shuttle to the cytoplasm in complex with mRNA (Piñol-Roma and Dreyfuss, 1992, 1993). hnRNP C1/C2 can bind both the 3′- and 5′-termini of poliovirus negative-sense RNA intermediates (regions complementary to the 5′NCR and 3′NCR of genomic RNA, respectively) and has been proposed to play a role in poliovirus RNA replication by facilitating and/or stabilizing the terminal strand separation required for replication of this template (Roehl and Semler, 1995; Brunner et al., 2005; Ertel et al., 2010). The association of hnRNP C1/C2 with both termini of the negative-sense RNA molecule may also allow for the end-to-end linkage of this RNA template via the multimerization of hnRNP C1/C2 tetramers, since the multimerization domain of this protein is required for efficient *in vitro* replication of poliovirus RNA (Ertel et al., 2010). Recombinant hnRNP C1/C2 is able to rescue positive-strand RNA synthesis in cellular extracts depleted of endogenous hnRNP C1/C2, supporting a critical role for this protein in the production of poliovirus genomic RNA (Brunner et al., 2005). It has also been demonstrated that hnRNP C1/C2 interacts with the poliovirus protein 3CD (the polymerase precursor) through glutathione S-transferase pull-down assays; therefore, hnRNP C1/C2 may aid in the recruitment of the 3D polymerase to the replication template (Brunner et al., 2005). Furthermore, reduced cellular levels of hnRNP C1/C2 cause a decrease in the kinetics of poliovirus RNA synthesis during infection (Brunner et al., 2010). Combined with the finding that an intact 3′ NCR of poliovirus genomic RNA contributes to positive-strand RNA synthesis efficiency through complementary elements conserved at the 5′ end of negative-sense strand, a model for positive-sense RNA synthesis has been proposed. This model is reminiscent of negative-sense RNA production, with both ends of the RNA template in close proximity, albeit in the form of a predominantly dsRNA molecule (Ertel et al., 2010). Due to the proximity of the ternary complex found on the 5′-end of the positive-sense poliovirus RNA molecule, it is possible that at least one polymerase utilized in the synthesis of the positive-sense RNA is recruited directly from the ternary complex on the genomic RNA molecule to the negative-sense template, as suggested by the trans-initiation model (Vogt and Andino, 2010) (Figure 4B).

The circularization of RNA templates proposed to promote poliovirus RNA replication is made possible by nuclear-resident proteins. Circularized templates may serve to function as a fidelity check on the RNA itself, to act *in lieu* of a true promoter region to enhance the initiation of RNA synthesis, and to provide a mechanism by which 3D specifically recognizes a polyadenylated mRNA of viral origin in an infected cell containing abundant polyadenylated mRNA transcripts. While there has been little exploration of the RNA replication process in genera beyond the enteroviruses, it is possible that, as with the handful of nuclear proteins that are considered picornavirus-general ITAFs, there is a minimal requirement of particular nuclear-resident proteins to promote picornavirus RNA replication (at present the three major players appear to be PCBP2, PABP1, and hnRNP C1/C2), with other cellular proteins that may be species-specific that act to further enhance the efficiency of RNA replication. As with translation, the cohort of proteins utilized for picornavirus RNA replication may also be dependent upon the availability of particular proteins within the context of the cellular microenvironment where the viral RNA molecules are located. The different levels of enhancement provided to picornavirus RNA replication through the activities of different (or available) nuclear-resident host-cell proteins may contribute to the variable ratios of positive- to negative-sense RNA ratios, which have been reported to range from 30:1 to 70:1 for poliovirus (Andino et al., 1990; Giachetti and Semler, 1991; Novak and Kirkegaard, 1991).

## Alteration to nucleocytoplasmic trafficking causes the loss of normal subcellular localization of nuclear-resident proteins and facilitates picornavirus replication

As discussed previously, the initial rounds of picornavirus translation and RNA replication are dependent on nuclear-resident proteins that are present in the cytoplasm of the infected cell as a result of their shuttling function or nascent biogenesis. As the replication process continues, there is an amplification in both viral protein production and RNA replication as the number of viral RNA templates grows. The low concentration of nuclear proteins normally present in the cytoplasm is no longer sufficient to meet the increased demand for these proteins. As a result, picornaviruses alter nuclear-cytoplasmic trafficking to provide the functions of normally nuclear-resident proteins to the cytoplasm where viral replication takes place.

### Enterovirus proteinases degrade the nucleoporin proteins of the NPC

Because the NPC is the main route by which the nucleus and cytoplasm exchange material, picornaviruses target the NPC specifically to disrupt normal protein trafficking pathways resulting in the cytoplasmic accumulation of cellular proteins. Enterovirus (poliovirus or HRV 14) infection alters both the classical import pathway, which relies on a heterodimer consisting of an importin-α (karyopherin α) adaptor protein that binds the arginine-lysine-rich NLS of the cargo protein and transport receptor importin-β1 (karyopherin β1), as well as the transportin-1 (karyopherin β2) pathway in which the import receptor transportin recognizes a glycine-rich motif known as the M9 NLS (reviewed in Cautain et al., 2015). In uninfected cells expressing enhanced green fluorescent protein (EGFP) linked to either a classical NLS derived from the large T antigen of simian virus 40 (SV40) or the M9 NLS of hnRNP A1, EGFP localizes to the nucleus. However, upon infection with poliovirus, an accumulation of EGFP protein in the cytoplasm is observed by 4.5 h post-infection, demonstrating a disruption in these two import pathways as a consequence of infection (Gustin and Sarnow, 2001). Additionally, hnRNP K, which contains a unique 40-amino acid motif NLS, known as KNS, is also relocalized to the cytoplasm of poliovirus infected cells, suggesting the import of proteins through the KNS-mediated pathway is also prevented during infection (Michael et al., 1997; Gustin and Sarnow, 2001). HRV 14 infection also causes cytoplasmic localization of EGFP fusion proteins containing a classical or M9 NLS, albeit at later times during infection than observed for poliovirus (Gustin and Sarnow, 2002). Conversely, an EGFP fusion protein containing an NLS that mediates nuclear import through a hormone-dependent but unknown importin-α-independent pathway remains localized to the nucleus upon poliovirus infection, suggesting that specific import pathways are targeted by poliovirus, while some import pathways remain functional. Furthermore, an EGFP fusion protein containing a leucine-rich NES recognized by the chromosome region maintenance 1 (CRM1) export receptor is localized to the cytoplasm during infection, and a small molecule inhibitor of CRM1 causes retention of the EGFP fusion protein in the nucleus, suggesting that this export pathway is unaltered in poliovirus infected cells (Gustin and Sarnow, 2001).

Like poliovirus, CVB3 also causes the relocalization of GFP fused with a classical NLS, indicating that particular alterations to nuclear-cytoplasmic transport pathways is a general feature of enteroviruses (Belov et al., 2000). However, the use of Timer proteins that change emission fluorescence based on their age has shown that the accumulation of nuclear proteins in the cytoplasm of enterovirus infected cells is the result of increased efflux of these proteins from the nucleus as a result of NPC degradation, rather than simply disruption of the import of newly-synthesized proteins into the nucleus (Belov et al., 2004).

The finding that nuclear-resident proteins accumulate in the cytoplasm of cells upon picornavirus infection as a result of increased protein efflux from the nucleus appears at odds with the observation that picornavirus-induced disruptions in nuclear import cause cytoplasmic retention of newly synthesized nuclear-resident proteins. However, these seemingly disparate findings can be reconciled upon closer examination of the particular alterations made to the NPC during infection. The increased permeability of, and inability to import proteins through the NPC during enterovirus infection is the result of changes made to Nup proteins that comprise the NPC itself. Electron microscopy of poliovirus-infected cells shows structural alterations to the nuclear envelopes and nuclear pores, specifically the loss of an obstructing bar-like structure in the central channel, which is at least partially caused by the viral proteinase 2A. General inhibitors of the 2A proteinase suppress the efflux of marker proteins from the nucleus during infection. In addition, transfection of a wild type 2A expression construct, but not a construct encoding an inactive 2A, into cells yields cytoplasmic re-localization of stably expressed GFP-NLS proteins (Belov et al., 2004). Like enteroviruses, the cardiovirus EMCV breaks down the specificity of bidirectional protein traffic through the NPC in infected cells by directly modifying the architecture of the NPC (Lidsky et al., 2006).

Structural data from electron microscopy studies showing destruction of the NPC and products of proteolysis within the pore bolster biochemical data that demonstrates the degradation of Nup 153 and Nup 62 in cells infected with either poliovirus or rhinovirus. Moreover, immunofluorescence microscopy indicates a decrease in overall levels of these Nups as the course of an enterovirus infection proceeds (Gustin and Sarnow, 2001, 2002). Prior to the proteolysis of Nup 153 and Nup 62, Nup 98 is degraded by 2A in poliovirus-infected cells. The cleavage of Nup 98 is insensitive to guanidine hydrochloride treatment, which inhibits enterovirus RNA replication and results in reduced viral protein production, whereas the cleavage of Nup 153 and Nup 62 is sensitive to the presence of guanidine. In conjunction with the fast kinetics of this cleavage (within 1 h post-infection), this suggests that Nup 98 is cleaved even when there is a very low concentration of viral protein present within the infected cell. This also suggests that poliovirus (and perhaps other enteroviruses) may target specific Nups and trafficking pathways at different times in the infectious cycle to facilitate viral replication (Park et al., 2008). The addition of purified HRV 2 2A to whole cell lysates causes the cleavage of Nup 98 while the expression of poliovirus 2A in cells results in the degradation of Nup 62, Nup 98, and Nup 153, demonstrating that 2A is able to alter components of the NPC (Park et al., 2008; Castelló et al., 2009). Furthermore, purified HRV 2 2A is able to cleave recombinant Nup 62 *in vitro*. This cleavage event liberates an FG-rich region, a domain important for nuclear transport receptor association during transport through the NPC, of Nup 62 during infection (Park et al., 2010). Interestingly, experiments utilizing recombinant 2A from the three different HRV clades demonstrated that these proteinases cleave Nup 62, Nup 98, and Nup 153 at distinct sites and with variable rates (Watters and Palmenberg, 2011).

Enterovirus 2A has an obvious role in nucleoporin proteolysis during infection; however, two additional nucleoporins, Nup 214 and Nup 358, as well as the previously mentioned Nup 153, are degraded in cells transfected with HRV 16 3C or 3CD expression constructs, suggesting that the proteolytic activity of 2A alone does not account for all Nup degradation in enterovirus-infected cells (Ghildyal et al., 2009). In support of this suggestion, Nup 62 does not contain a 2A specific Tyr-Gly cleavage site and the size of Nup 153 cleavage products from poliovirus-infected cells do not correspond to those expected if 2A does degrade this nucleoporin (Belov et al., 2004). Moreover, high concentrations of purified HRV 14 3C are able to induce the partial cleavage of Nup 62 *in vitro* (Park et al., 2010). Enterovirus-induced alterations to the NPC cause the loss of normal protein partitioning not only for nuclear-resident proteins but also for cytoplasmic-resident proteins, demonstrated by the distribution of normally cytoplasmic-resident proteins such as GAPDH and cyclin-B1 throughout the cytoplasm and nucleus of enterovirus-infected cells (Belov et al., 2004).

### Cardiovirus infection induces the hyper-phosphorylation of nucleoporin proteins

The 2A proteins of cardioviruses lack proteinase activity, and Nup 62 as well as Nup 153 are stable in mengovirus infected cells (Lidsky et al., 2006). Despite the lack of degradation of these nucleoporins, mengovirus and EMCV promote the redistribution of stably-expressed EGFP proteins containing the classical NLS of the SV40 large T antigen to the cytoplasm and normally cytoplasmic-resident proteins such as cyclin-B1 to the nucleus (Lidsky et al., 2006). The normal subcellular partitioning of proteins in cardiovirus-infected cells, like that of enterovirus-infected cells, is disrupted by dysregulation of bidirectional nucleocytoplasmic trafficking. Unlike enteroviruses, however, cardioviruses achieve this dysregulation through the action of the leader (L) protein. Mengovirus mutants lacking the leader protein coding sequence or encoding an L protein with a mutated zinc finger domain are unable to trigger the cytoplasmic redistribution of a stably-expressed GFP-NLS fusion protein in cells. Furthermore, phosphorylation of a threonine residue at position 47 of the L-protein of mengovirus has also been suggested to play a functional role in L-dependent alterations to nuclear-resident protein localization (Lidsky et al., 2006). Mutations to the L protein of TMEV, specifically disruptions made to the zinc-finger domain of this protein, also fail to facilitate the relocalization of endogenous nuclear proteins to the cytoplasm that are relocalized during infection with wild type TMEV (Delhaye et al., 2004). Protease inhibitors fail to suppress the cytoplasmic redistribution of stably expressed EGFP-NLS fusion proteins in EMCV-infected cellular extracts. Additionally, EMCV replicons containing mutations to the 2A coding sequence do not affect the nuclear envelope leakiness observed during EMCV infection. Taken together, these studies demonstrate that cardioviruses do not utilize a proteinase or viral protein 2A to promote alterations to nucleocytoplasmic trafficking.

In the absence of other cardioviral proteins, recombinant EMCV L alone is able to disrupt normal nuclear localization of a transiently transfected GFP-NLS, and an intact zinc-finger domain within the L protein is specifically required for the observed increase in permeability of the nuclear envelope. A cellular phosphorylation pathway is also required to induce nuclear envelope leakiness, because L protein does not possess kinase activity. Additionally, the protein kinase inhibitor staurosporine can rescue nuclear import/export activity from L-dependent inhibition (Porter and Palmenberg, 2009). Nup 62, Nup 153, and Nup 214 each become hyperphosphorylated in an L-dependent manner as shown by phosphoprotein staining during infection with EMCV, and similarly, Nup 62 and Nup 98 are hyperphosphorylated in mengovirus and TMEV infected cells, respectively, as shown by assaying for gel migration shifts following alkaline phosphatase treatment (Bardina et al., 2009; Porter and Palmenberg, 2009; Ricour et al., 2009b). The phosphorylation of Nup 62 and 98 in mengovirus and TMEV infected cells, respectively, is also dependent upon the zinc-finger domain of L (Bardina et al., 2009; Ricour et al., 2009b). Interestingly, there is no phosphorylation level change of Nup 358 during cardiovirus infection, a Nup that is cleaved during enterovirus infection, (Porter and Palmenberg, 2009).

Direct architectural changes to the NPC can be observed through electron microscopy of NPC cross-sections from cardiovirus infected cells. The central channel of nuclear pores is less electron dense in infected compared to uninfected cells, similar to what is observed in poliovirus-infected cells. How phosphorylation of Nups achieves un-blocking of these pores is not clear (Lidsky et al., 2006; Bardina et al., 2009). One possibility is that the increased negative charge present on FG-motif containing fibrils, as a result of phosphorylation, within the pores that normally act to block passive diffusion of macromolecules, could promote retraction of fibrils to the NPC scaffold leaving the pore empty (Cohen et al., 2012). Through screening a panel of kinase inhibitors, Nup hyperphosphorylation appears to be carried out via two mitotic terminal kinase effectors within the mitogen activated protein kinase cascade: extracellular signal-regulated receptor kinase (ERK) and p38 mitogen-activated protein kinase (p38), although the exact mechanism by which L co-opts these kinases is not known (Porter et al., 2010). A C-terminal acidic domain within the L protein is also important for the Nup hyperphosphorylation, perhaps via interactions with MAPK pathway regulatory proteins (Porter et al., 2010). Another kinase that may have a role in L-dependent alterations to nucleocytoplasmic trafficking is casein kinase II (CK-2), as it has been shown to phosphorylate Thr-47 of mengovirus L, a phosphorylation event that has a functional role in nuclear-protein efflux (Zoll et al., 2002; Lidsky et al., 2006). The L protein of EMCV has also been shown to bind directly to Ran-GTPase, the concentration of which provides the gradient that imparts directionality to transport, suggesting cardioviruses may utilize multiple strategies to inhibit homeostatic nucleocytoplasmic trafficking (Porter et al., 2006). mRNA export has also been reported to be inhibited in cells expressing TMEV L protein by assaying for poly(A) transcript retention in the nucleus of cells via *in situ* hybridization (Ricour et al., 2009b).

### FMDV infection does not cause dysregulation of nucleocytoplasmic trafficking

Interestingly, another picornavirus of the aphthovirus genus, FMDV, which encodes a proteolytic L-protein, does not appear to target nucleoporins for degradation (Castelló et al., 2009). Moreover, infection with FMDV has not been reported to alter general nucleocytoplasmic trafficking, although some nuclear-resident proteins are redistributed to the cytoplasm of infected cells, likely through a more cellular protein-specific directed approach (Lawrence and Rieder, 2009; Lawrence et al., 2012).

### Alterations to NPC components are not a result of apoptosis

Apoptotic cell death has been shown to cause damage to the nuclear envelope barrier, including cleavage of Nup 153, through the actions of cellular caspase-9 (Buendia et al., 1999; Faleiro and Lazebnik, 2000). Because enteroviruses can promote apoptotic cell death, it is theoretically possible that the increases in NPC permeability observed during picornavirus infection are due to caspase-9 induction rather than the direct actions of viral proteins themselves. Indeed, poliovirus can cause the initiation of an apoptotic program through caspase-9, and expression of 2A alone can cause cell death through apoptosis (Tolskaya et al., 1995; Agol et al., 1998; Goldstaub et al., 2000; Belov et al., 2003). However, cells deficient in caspase-9 (as well as associated caspase-3) did not show differences in NPC permeability compared to cells expressing normal levels of caspases when infected with poliovirus, suggesting that the increased permeability of the NPC during picornavirus infection is independent of the action of pro-apoptotic caspases (Belov et al., 2004). Moreover, Nup 62 destruction is a marker of picornavirus-infected cells but not apoptotic cells (Buendia et al., 1999; Gustin and Sarnow, 2001, 2002). HAV, CVB3, and TMEV induce apoptosis as well, but these same picornaviruses can also inhibit apoptosis, indicating that the consistent relocalization of cellular proteins to different cellular compartments seen during infection is likely not attributable to pathways involved in programmed cell death (Tolskaya et al., 1995; Gosert et al., 2000b; Henke et al., 2001; Jelachich and Lipton, 2001; Neznanov et al., 2001; Belov et al., 2003; Romanova et al., 2009).

Although both increased efflux of nuclear resident proteins as a result of the dysregulation of the barrier function of the nuclear envelope as well as impediments to nuclear import of nascently produced cellular proteins in the cytoplasm (i.e., prior to host protein translation inhibition by viral infection) have been demonstrated to occur in picornavirus infected cells, these mechanisms are not mutually exclusive. Enterovirus-induced cleavage of Nups 62, 98, 153, 214, and 358 by the 2A and 3CD/3C proteinases, or cardiovirus-induced hyper-phosphorylation of Nups 62, 98, 153, and 214 through actions of the Leader protein, is directly responsible for both the increased “leakiness” of the nuclear envelope and the lack of nuclear import receptor–cargo complex docking at the cytoplasmic face of the NPC. Nucleocytoplasmic trafficking is an intricate and tightly regulated process allowing for precise control of gene expression. As a result, drastic alterations to the components of the NPC gateway between the two major compartments of the eukaryotic cell will have diverse and far-reaching consequences on trafficking pathways. The picornaviruses have evolved to take advantage of this important regulatory node to provide the functions of nuclear-resident proteins to the cytoplasm to promote their replication.

### Picornavirus induced alterations to nucleoporin proteins result in the redistribution of cellular proteins

**Figure 5:**
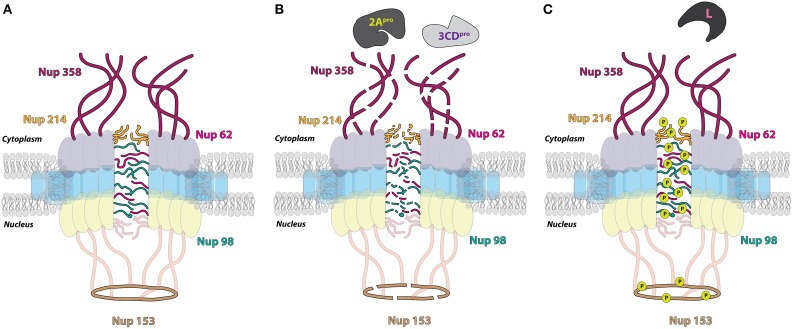
**Picornavirus-induced alterations to the nuclear pore complex (NPC). (A)** Regions of the NPC where the five nucleoporin (Nup) proteins targeted by picornavirus proteins during infection are located. **(B)** Enterovirus proteinase 3CD/3C (3CD^pro^) cleaves Nup358 (fuschia) and Nup153 (brown), which are components of the cytoplasmic filaments and nuclear basket, respectively. The 2A proteinase (2A^pro^) of enteroviruses degrades FG-containing barrier Nups of the central channel, including the cytoplasmic Nup214 (yellow), as well as Nup62 and Nup98 (pink and green, respectively). **(C)** Cardiovirus infection induces the hyper-phosphorylation (P within chartreuse circle) of the same Nups targeted by enteroviruses, excluding Nup358, through the actions of the Leader protein (L).

The five Nup proteins that are the targets of alteration by picornavirus proteins have critical roles in shuttling macromolecules through the NPC (Figure 5). Nup 214 and 358 are positioned on the cytoplasmic side of the NPC, with Nup 358 making up cytoplasmic filaments of the NPC, and Nup 214 residing on the cytoplasmic face of the NPC. Nup 62 is localized to the central pore of the NPC and Nup 153 is a component of the nuclear basket. Nup 98 is found within and on both sides of the NPC and can function, to some degree, independently of the NPC due to its mobile nature (Griffis et al., 2002). All five of these nucleoporins contain FG-repeat domains, indicative of their direct role in nucleocytoplasmic transport (reviewed in Chatel and Fahrenkrog, 2011). Nup 62, in association with other Nups, forms a central “plug” in the channel of the NPC, and the cleavage of this protein by enteroviral proteinases has been suggested to account for the loss of the electron-dense material within the NPC and appearance of “granules” likely corresponding to Nup cleavage products, as observed via electron microscopy (Bardina et al., 2009). This particular alteration could allow for the diffusion of proteins in and out of the nucleus, accounting for the increased “leakiness” of the nuclear envelope observed as a result of infection (Belov et al., 2004). Interestingly, it appears that the alterations made to these proteins individually are not sufficient to promote a loss of normal cellular protein partitioning and that inhibition of nuclear import can only occur in cells with a composite of nucleoporin alterations (Park et al., 2008). Nup 153 has been reported to interact with importin α/β and transportin; therefore, alterations to Nup 153 are at least partially able to account for the inhibition of the nucleocytoplasmic trafficking pathways that rely on these receptors (Shah et al., 1998; Nakielny et al., 1999). Although Nup 98 has been implicated in mRNA export from the nucleus, alterations made to this nucleoporin during enterovirus infection do not inhibit cellular mRNA export, in contrast to the mRNA export inhibition observed in cardiovirus infected cells (Powers et al., 1997; Griffis et al., 2003; Porter et al., 2006; Park et al., 2008; Ricour et al., 2009b). Notably, however, a study in which an enteroviral 2A expression construct was electroporated into cells demonstrated that nuclear export of mRNAs, U snRNAs, and rRNAs but not tRNAs was blocked when 2A was expressed (Castelló et al., 2009). In addition to FG domains present in Nup 358 and Nup 153, these nucleoporins also contain Ran binding domains and as a result, picornavirus targeting of these proteins could conceivably have profound consequences on the compartmentalization of cellular proteins by disruption of the Ran gradient (Wente and Rout, 2010). The L-protein of EMCV, and likely cardioviruses in general, is simultaneously able to disrupt the differential Ran gradient across the nuclear envelope by directly binding and sequestering Ran (Porter et al., 2006).

The picornavirus-mediated changes to components of the NPC critical to nucleocytoplasmic trafficking increase the bidirectional transport of proteins within the infected cell, thus causing a massive dysregulation of the transport process as a whole. However, there appears to be some level of specificity to which import and export pathways are affected, as a subset of nuclear-resident proteins relocalize while others do not. For example, nuclear-resident proteins fibrillarin, TATA-box-binding-protein 1 (TBP1), and serine/arginine-rich splicing factor 2 (SRSF2 or SC35) do not relocalize from the nucleus to the cytoplasm during poliovirus infection (Meerovitch et al., 1993; McBride et al., 1996; Waggoner and Sarnow, 1998; Gustin and Sarnow, 2001). SRSF2 is imported into the nucleus through a unique import receptor called transportin-SR. So it is possible that this pathway is unaffected by picornavirus infection (Kataoka et al., 1999). However, because picornavirus infection halts cellular translation in the early stages of infection, the persistence of some nuclear-resident proteins in the nucleus cannot be attributed to continued transport of newly synthesized proteins to this subcellular compartment. Nuclear-shuttling proteins, however, could be inhibited from re-entering the nucleus by picornavirus-mediated disruptions to trafficking pathways, trapping these proteins in the cytoplasm. Protein-specific nuclear retention signals (NRSs) may allow some proteins to maintain normal localization throughout the course of infection, although not all proteins that contain NRSs remain in the nucleus, as even the strongly nuclear-resident hnRNP C1/C2 is relocalized to the cytoplasm between 3 and 4.5 h following infection with enteroviruses (Gustin and Sarnow, 2001). Further complicating attempts to fully explain how picornavirus infection affects the subcellular localization of endogenous proteins is the fact that some normally-nuclear-resident proteins such as nucleolin, La, Sam68, hnRNP A1, hnRNP K, and hnRNP C1/C2 are almost completely relocalized to the cytoplasm upon poliovirus infection (as observed by immunofluorescence microscopy), even though a general increase in bidirectional trafficking through the nuclear envelope would predict a uniform distribution of these proteins between the nucleus and cytoplasm (Gustin and Sarnow, 2001). It is possible that the apparent uneven redistribution of some proteins between the nucleus and cytoplasm as a result of picornavirus infection can be interpreted as these proteins being associated with specific cellular or viral structures that do not change their subcellular location upon infection. For example, retention of nuclear-resident proteins within the nucleus following infection could be explained by these proteins having strong associations with DNA, which remains in the nucleus regardless of virus-induced nuclear pore modifications.

Many proteins, including nucleolin, hnRNP A1, PTBP1, La, Sam68, hnRNP K, hnRNP C1/C2, SRSF3, and TDP2, that are relocalized as a result of infection have known functions in the replication cycle of picornaviruses (Meerovitch et al., 1993; McBride et al., 1996; Waggoner and Sarnow, 1998; Gustin and Sarnow, 2001, 2002; Back et al., 2002; Ricour et al., 2009a; Fitzgerald and Semler, 2011; Virgen-Slane et al., 2012; Fitzgerald et al., 2013). Aside from direct roles in picornavirus replication, redistribution of nuclear proteins likely has consequences on cellular homeostasis. For example, because Sam68 functions in cell cycle transitions, the redistribution of this protein as a result of infection could disrupt the cell cycle. Other proteins, including various splicing factors which appear to have no role in viral replication, have also been shown to redistribute to the cytoplasm of poliovirus 2A expressing cells, likely as a mechanism to further disrupt host-cell gene expression (Alvarez et al., 2013). While the relocalization of most cellular proteins utilized by picornaviruses during infection can be attributed to disruption of homeostatic nucleocytoplasmic trafficking, some proteins are relocalized, at least partially, through direct cleavage by viral proteinases and the subsequent loss or alteration of functional NLS regions within these proteins. Cleavage of PTBP1 and La, both of which contain a bipartite NLS, by poliovirus 3CD/3C exemplifies this phenomenon (Simons et al., 1996; Romanelli et al., 1997; Shiroki et al., 1999; Back et al., 2002). RHA and Sam68, which likely function in aphthovirus RNA replication and translation, respectively, are mislocalized in FMDV infected cells. This relocalization has been attributed to the demethylation of RHA and liberation of NLS containing-regions of Sam68 through cleavage by FMDV L proteinase (Lawrence and Rieder, 2009; Lawrence et al., 2012). Furthermore, the nuclear efflux of RHA occurs with a concomitant influx of Jumonji C-domain containing protein 6, a demethylating protein that may act on RHA. This suggests that because FMDV does not degrade nucleoporin proteins directly, it may have more precise control over cellular protein trafficking compared to general disruptions in trafficking pathways observed with other picornaviruses (Lawrence et al., 2014).

### Virus-induced NPC alterations lead to interference with host antiviral defenses

In addition to concentrating proteins with functions that are co-opted by picornaviruses for replication in the cytoplasm, alterations to nucleocytoplasmic trafficking can also disable innate antiviral signaling cascades in infected cells. One of the earliest host responses to viral infection, the innate antiviral response, is mediated primarily by the action of Type-I interferons (IFNs). This response is activated by the cellular recognition of viral molecules called pathogen associated molecular patterns (PAMPs). One major receptor of intracellular PAMPs that recognize picornavirus family members is interferon-induced helicase C domain-containing protein 1 (also known as melanoma differentiation-associated protein 5 or MDA-5) (Kato et al., 2006). Upon PAMP recognition, cytoplasmic receptors such as MDA-5 initiate a cascade of events that results in the activation of transcription factors such as IFN regulatory factor 3 (IRF-3), nuclear factor (NF)-κ-B, and transcription factor AP-1. Importins then act in transporting these transcription factors through the NPC to the nucleus, which, in turn, activates the transcription of type I interferons (IFN-α/β) and interferon-stimulated genes (ISGs). The resultant IFN mRNA is exported to the cytoplasm where it is translated and then secreted, inducing a secondary response in an autocrine and paracrine process. This results in the activation of a second signaling cascade involving many effectors and transcription factors such as signal transducer and activator of transcription (STAT) proteins, which translocate to the nucleus. Within the nucleus, these proteins activate transcription of additional ISGs. These IFN stimulated gene products target the pathogen for destruction (Younessi et al., 2012).

Changes made to the NPC by picornaviruses can lead to an attenuation in ISG expression and the associated antiviral response. Nup 98 is cleaved much more rapidly than the other Nup targets in poliovirus infected cells, and this has been suggested to be an early target of picornavirus proteins to diminish the transcriptional-induction of antiviral genes. Experiments utilizing guanidine hydrochloride, which inhibits enterovirus RNA replication and, as a result, normal levels of viral protein production, have shown that cleavage of Nup 98 in poliovirus-infected cells is not sufficient to cause relocalization of proteins like nucleolin and that high concentrations of viral proteins are needed to degrade other nucleoporin targets to allow the widespread redistribution of nuclear-resident proteins to the cytoplasm (Park et al., 2008). Nup 98 is a component of the NPC but can also be found dissociated from this complex both in the nucleus and the cytoplasm. Enteroviral 2A and cardioviral L may be capable of targeting free Nup 98 prior to direct transcriptional shut-off, which occurs when 3C precursors enter the nucleus of infected cells (discussed in the subsequent section), and interfere with signaling to the nucleus and/or the export of ISG transcripts. As a result, the virus may be able to avoid inducing an early antiviral response within the cell and promote maximal viral amplification (Park et al., 2008). Interestingly, Nup 98 has recently been shown to act as a transcription factor and induce the expression of antiviral defense genes in *Drosophila* (Panda et al., 2014). Therefore, the rapid degradation of Nup 98 in infected cells could allow enteroviruses to limit innate antiviral responses as well as set the stage for dysregulation of nucleocytoplasmic trafficking early during infection. The importance of restricting the production of IFN-α/β during picornavirus infection is demonstrated by the fact that pre-treatment of cells with IFN-α/β inhibits picornavirus replication, confirming that once ISG products are expressed they cannot be overcome (Chinsangaram et al., 2001).

As mentioned above, the cardioviral TMEV L protein prevents export of cellular mRNAs from the nucleus. Furthermore, the TMEV L protein inhibits the transcription of cytokine and chemokine genes that are ordinarily activated upon viral infection (Van Pesch et al., 2001). This inhibition can be attributed to the fact that TMEV infection disallows the formation of IRF-3 dimers, which normally translocate to the nucleus to regulate transcription of antiviral genes (IFN-α/β as well as ISGs) in response to infection. Because infection with TMEV containing a mutation in the zinc finger motif of L or a partial deletion of L allows the dimerization of IRF-3 but wild type TMEV infection does not, the inability of this nuclear translocation and subsequent antiviral protein production can be attributed to antagonizing this pathway by the L protein. Moreover, disruptions in nucleocytoplasmic trafficking and the block to mRNA export from the nucleus correlate with Nup 98 hyper-phosphorylation (Ricour et al., 2009a). The leader protein of mengovirus has also been demonstrated to inhibit IFN-α/β expression in infected cells by suppressing the activation of NF-κB, a suppression dependent upon phosphorylation of Thr-47 in the L protein (Zoll et al., 2002). Interestingly, negative-sense RNA complementary to the L coding region of the EMCV genome was recently shown to be a determinant of MDA-5 mediated interferon production (Deddouche et al., 2014). Finally, the L proteinase of FMDV functions in the inhibition of IFN-β mRNA induction. However, this inhibition is not dependent upon the dysregulation of cellular nucleocytoplasmic trafficking because FMDV does not target these pathways. Instead, the FMDV L proteinase enters the nucleus and promotes the degradation of an NF-κB component (De Los Santos et al., 2006, 2007).

### The redistribution of cellular proteins as a result of infection is not necessarily specific

Because fundamental components and regulatory mechanisms of nucleocytoplasmic trafficking are disrupted during picornavirus infection, some proteins that relocalize to the cytoplasm of infected cells have no known function in viral replication, and in some instances even have antiviral roles. AU-rich binding factor 1 (AUF1, also known as hnRNP D0) binds with high affinity to RNA molecules containing AU-rich elements (usually in the 3′ non-translated region), is a predominantly nuclear-resident protein that shuttles to the cytoplasm, and promotes mRNA turnover (reviewed in Gratacós and Brewer, 2010). AUF1 relocalizes from the nucleus to the cytoplasm of poliovirus, HRV 14, and HRV 16 infected cells in a 2A-driven manner and binds to S-L IV within the IRES of poliovirus RNA (Rozovics et al., 2012; Cathcart et al., 2013). The presence of this protein inhibits poliovirus translation in a dose dependent manner *in vitro*, and cells lacking AUF1 produce higher titers of poliovirus, CVB3, and HRV 1a (Cathcart et al., 2013). The enteroviral proteinase precursor 3CD and mature 3C cleave AUF1 *in vitro*, leading to a decrease in the affinity of AUF1 for poliovirus S-L IV (Cathcart et al., 2013; Wong et al., 2013). Consequently, enteroviral infection and resultant NPC degradation cause the relocalization of this negative regulator of viral replication, but subsequent action by viral proteinases diminishes the antiviral effect of AUF1. Similar results have been observed with EV71 (Lin et al., 2014). Interestingly, AUF1 also relocalizes from the nucleus during cardiovirus infection but does not have a negative effect on EMCV amplification and is not degraded during infection (Cathcart and Semler, 2014). The fact that the anti-enteroviral AUF1 is relocalized in enterovirus-infected cells demonstrates that nucleocytoplasmic trafficking disturbances can cause relocalization of a broad subset of nuclear-resident proteins that do not necessarily benefit viral replication.

Although cardioviruses and enteroviruses target many of the same nucleoporin proteins, the mechanisms that cause cytoplasmic accumulation of nuclear proteins are distinct. Enteroviral proteinases degrade FG-repeat containing Nups while cardioviruses promote phosphorylation of these Nups. Nonetheless, the consequences of Nup alterations by picornaviruses are equivalent: they provide the proteins that facilitate viral replication in the cytoplasm. Additionally, degradation of NPC components functions as a mechanism to limit signal transduction to the nucleus and thereby attenuate host antiviral defense pathways. Although to what degree picornaviruses are able to specifically orchestrate different nucleocytoplasmic trafficking pathways is unclear, increased NPC leakiness (i.e., increased export of outbound macromolecules from the nucleus and the restriction of inbound cargo from the cytoplasm) is a functionally significant event that many picornaviruses have evolved to accomplish in order to mount effective cytoplasmic replication strategies.

## Picornavirus proteins enter the nucleus to limit host-cell gene expression

In addition to shutting down most host-cell translation through actions in the cytoplasm, some picornaviruses also target the transcriptional components of cellular gene expression. Picornavirus infection has long been known to greatly reduce the initiation rate of cellular RNA synthesis (Baltimore and Franklin, 1962). This occurs despite the fact that cellular DNA-dependent RNA polymerase II (Pol II) itself is functional during infection. However, at least one factor required for Pol II transcription is deficient in picornavirus-infected cells (Apriletti and Penhoet, 1978; Crawford et al., 1981). Viral protein synthesis is required to induce this cellular transcription inhibition, and cytoplasmic extracts of poliovirus-infected cells inhibit RNA synthesis in isolated nuclei (Franklin and Baltimore, 1962; Balandin and Franklin, 1964; Bossart et al., 1982). This early work prompted the question of whether picornavirus proteins are able to enter the infected cell nucleus to carry out this inhibitory task. Subsequent studies demonstrated that radiolabeled viral proteins do in fact enter the cell nucleus (Bienz et al., 1982; Fernández-Tomás, 1982).

### Picornavirus infection results in the termination of cellular transcription

Enterovirus infection interferes with transcription driven by all three DNA-dependent RNA polymerases of mammalian cells. RNA polymerase I (Pol I) synthesizes ribosomal RNA (except 5S rRNA), which accounts for over half the RNA produced in the cell, and is carried out in the nucleolus (Russell and Zomerdijk, 2006). The Pol I transcription machinery is made up of two major transcription factor complexes: upstream binding factor (UBF) and selectivity factor 1 (SL1). Poliovirus infection was first shown to alter Pol I driven transcription using *in vitro* transcription systems in combination with uninfected or poliovirus-infected cellular lysates. Oligonucleotides containing the Pol I promoter element in combination with poliovirus-infected HeLa cell extracts results in faster migrating complexes by electrophoretic mobility shift assays compared to the same oligonucleotides incubated with uninfected extracts. Incubation of poliovirus 3C with mock-infected extracts recapitulated this result, suggesting that this viral proteinase is responsible for cleavage of host factors required for Pol I driven transcription. This claim was verified when incubation of purified poliovirus 3C with cellular extracts was shown to produce near total inhibition of Pol I transcription (Rubinstein et al., 1992). The inhibition of Pol I transcription during poliovirus infection has been attributed to the inactivation of both UBF and SL1, two components of the Pol I transcription initiation complex. *In vitro* restoration of rRNA transcription requires the addition of both UBF and SL1 to extracts from poliovirus-infected cells. Poliovirus 3C cleaves TATA box-binding protein-associated factor RNA polymerase I subunit C (TAF 1C or TAF110), a component of SL1. Although UBF is not targeted by 3C *in vitro*, it does appear to be modified by a 3C-dependent mechanism within poliovirus infected cells (Banerjee et al., 2005).

The Pol II transcription system is responsible for the production of messenger RNA within eukaryotic cells. TFIID binding to the TATA box DNA sequence is the initial transcriptional step in the formation of the preinitiation complex and is performed by TBP1, a component of TFIID. TFIID activity in poliovirus-infected cells is greatly decreased compared to mock-infected cells and only TFIID is capable of restoring basal Pol II transcription *in vitro* (Kliewer and Dasgupta, 1988; Yalamanchili et al., 1996). The decrease in TFIID activity during poliovirus infection has also been attributed to 3C, as extracts from cells infected with a poliovirus encoding 3C with reduced activity is less effective at inhibiting Pol II transcription *in vitro*. Further experiments involving the co-transfection of constructs encoding poliovirus 3C and plasmids competent in Pol II transcription demonstrated that 3C is sufficient to inhibit Pol II transcription within cells (Yalamanchili et al., 1996). Poliovirus proteinases target TBP1 for cleavage during infection, and incubation of TBP1 with poliovirus 3C and 2A recapitulate the degradation of TBP1 *in vitro* (Clark et al., 1993; Das and Dasgupta, 1993; Yalamanchili et al., 1997a). However, only 3C is capable of inhibiting Pol II transcription within these cellular extracts. Furthermore, poliovirus inhibits activator-dependent Pol II transcription by cleaving and promoting the dephosphorylation of cyclic AMP-responsive element-binding protein 1 (CREB-1), leading to inhibition of CREB-1-activated transcription in poliovirus infected cells (Kliewer et al., 1990; Yalamanchili et al., 1997b). POU domain, class 2, transcription factor 1 (known as octamer-binding transcription factor 1 or Oct-1) is also specifically cleaved in poliovirus-infected cells and by 3C *in vitro*, and cleaved Oct-1 is unable to support activated transcription (Yalamanchili et al., 1997c).

Finally, the RNA polymerase III (Pol III) system, which controls the production of tRNA and 5S rRNA within eukaryotic cells, is also altered in poliovirus-infected cells. Studies similar to those discussed above demonstrate that 3C is responsible for cleaving the general transcription factor IIIC polypeptide 1 (TFIIIC box B-binding subunit or TFIIICα), leading to reduced activity of this transcription factor and subsequent inhibition of Pol III transcription (Fradkin et al., 1987; Clark and Dasgupta, 1990; Clark et al., 1991; Shen et al., 1996).

Although most work focusing on enterovirus-induced alterations to cellular transcription has implicated 3C, 2A also functions in this regard. Transient expression of poliovirus 2A in cells leads to reductions in DNA replication, Pol II transcription, as well as cap-dependent translation demonstrating that both enteroviral proteinases function in transcriptional repression (Davies et al., 1991). Probable ATP-dependent RNA helicase DDX20 (also known as Gemin3), a protein found in both the nucleus and cytoplasm of cells and which functions in biogenesis of spliceosomal complexes, is also proteolyzed in poliovirus-infected cells. The cleavage of this protein can be recapitulated *in vitro* using poliovirus 2A, and transfection of 2A results in Gemin3 cleavage in cells. Although the purpose of this cleavage event during infection is not clear, the assembly of spliceosomal complexes is reduced in infected cells and correlates with Gemin3 degradation and the loss of Gemin3 localization to the nucleus. Therefore, it is likely that cellular splicing is also targeted by enteroviruses during infection to further inhibit cellular gene expression (Almstead and Sarnow, 2007).

### Picornavirus proteins translocate to the nucleus of infected cells

To enter the nucleus of an infected cell and alter cellular transcription, poliovirus proteinase 3C utilizes an NLS present within the 3D polymerase domain and thus accesses the nucleus in the form of the 3CD precursor protein. The single, basic-type NLS present in 3D consists of amino acids 125–129, with the sequence KKKRD, which is a motif that is highly conserved within the enteroviruses. Interestingly, although 3D contains an NLS, this signal is necessary but not sufficient to allow the transport of 3CD into the nucleus. Constructs encoding poliovirus 3C, 3D, 3CD, or a mutated 3CD that is not capable of autoproteolysis, fused to EGFP, were transfected into cells and these cells were then infected with poliovirus or mock-infected. Despite the presence of the NLS in 3D, 3D-containing proteins were only relocalized to the nucleus of infected cells (Sharma et al., 2004). Mutation of the putative NLS sequence in 3D eliminates the nuclear localization of 3D-containing proteins, even in infected cells, demonstrating that the NLS as well as additional alterations to nucleocytoplasmic trafficking resulting from poliovirus infection are necessary for 3CD entry into the nucleus. Mutation of the 3D NLS results in cytoplasmic retention of transfected 3CD-EGFP expression constructs following infection, suggesting that this viral precursor protein does not enter the nucleus as a result of passive diffusion. Autoproteolysis of 3CD or trans-cleavage of one 3CD molecule by another, once within the nucleus, liberates 3C which then targets various transcription factors as discussed above. It should be noted that because 3CD is an active proteinase, this precursor form is also likely involved in the cleavage of host-cell proteins in the nucleus. Importantly, an NLS is required for 3CD to translocate to the nucleus, suggesting that the nuclear import pathway utilized by this viral precursor protein remains operational even in the face of the multiple alterations made to the NPC and nucleocytoplasmic trafficking in general during poliovirus infection. This provides further evidence that not all nuclear-resident proteins relocalize to the cytoplasm of poliovirus infected cells, including those which utilize transportin-SR import receptors (Gustin and Sarnow, 2001). The fact that the NLS of 3D most closely resembles a classical NLS while the import of proteins containing this type of NLS is altered in picornavirus-infected cells remains to be reconciled.

Recent evidence demonstrates that poliovirus 3D also functions in alterations of host cell gene expression within the nucleus. Once 3CD enters the nucleus and self-cleaves, 3D targets the pre-mRNA processing factor 8 (Prp8), a central component of the spliceosome, causing interference with pre-mRNA splicing and mRNA synthesis (Liu et al., 2014). Specifically, 3D associates with the C-terminal region of Prp8, resulting in lariat forms of the splicing intermediate accumulating within poliovirus infected cell nuclei.

In addition to inhibiting cellular transcription upon nuclear entry, poliovirus 3CD also targets components important for antiviral response and apoptotic pathways. Poliovirus infection initially leads to the activation of NF-κB, but the antiviral response is subsequently curtailed by 3C-mediated cleavage of the transcription factor p65 (p65/RelA) subunit of NF-κB (Neznanov et al., 2005). This alteration to cellular induction of antiviral pathways was shown to be independent of caspase-mediated cleavage, as the activation of caspase-3, the hallmark of apoptosis, was not observed in poliovirus-infected cells. Furthermore, the enteroviruses HRV 14 and Enteric Cytopathic Human Orphan virus-1 (ECHO virus-1), demonstrated a similar degradation of the NF-κB subunit p65/RelA. Poliovirus 3C also induces the degradation of the transcriptional activator and tumor suppressor protein p53 in a manner dependent upon the cellular protein promyelocytic leukemia protein (PML) during infection (Weidman et al., 2001; Pampin et al., 2006). Poliovirus infection regulates the intranuclear movement of PML from the nucleoplasm to the nuclear matrix, as well as recruitment of E3 ubiquitin-protein ligase MDM2, leading to the proteasome-dependent degradation of p53. This activity is hypothesized to counteract p53-target gene activation and ensuing induction of apoptosis (Pampin et al., 2006).

Studies focused on other enteroviruses have revealed similar insights into the translocation of viral proteins to the nucleus during infection. Cells infected with HRV 16, probed with antibodies against 3C, then imaged via confocal microscopy demonstrated that proteins containing HRV 16 3C accumulate in the nucleus of infected cells between 2 and 4 h post-infection, similarly to poliovirus 3C (Amineva et al., 2004). The NLS of HRV16 3D is likely located in the N-terminal portion of the polymerase, since a transfected RNA construct encoding 3D with a 371 amino acid deletion from the C-terminus is still able to localize to the nucleus. However, the nuclear localization of the truncated 3CD and full-length 3CD was observed in the absence of infection, contradicting previous work with poliovirus. Much like poliovirus, HRV 16 3CD degrades Oct-1 in infected cells (Yalamanchili et al., 1997c; Amineva et al., 2004). Cells infected with EV71 show decreased expression of cleavage stimulation factor subunit 2 (also known as CstF-64), which correlates, with the production of EV71 3C. Furthermore, 3C cleaves recombinant CstF-64 and inhibits cellular 3′-end pre-mRNA processing and polyadenylation *in vitro*. The accumulation of unprocessed pre-mRNA and reductions in mature mRNA are also observed in EV71-infected cells, suggesting that host-cell polyadenylation is targeted during EV71 infection (Weng et al., 2009).

Cardioviral proteins also enter the nuclei of infected cells. Indirect immunofluorescence and confocal microscopy revealed that 2A, VPg (3B), 3C, and 3D localize to the nucleus, and specifically to the nucleolus, of EMCV and mengovirus infected cells (Aminev et al., 2003a). Cardioviral 2A contains a short motif (KRVRPFRLP) that closely resembles an NLS found in yeast ribosomal proteins, and deletions within this sequence result in the loss of nucleolar localization (Svitkin et al., 1998; Aminev et al., 2003a; Groppo et al., 2011). Similar to observations of rhinoviral 3CD protein translocations to the nucleus, cardioviral 2A is capable of entering the nucleus in the absence of infection. Furthermore, cardioviral 2A colocalizes with nucleophosmin (nucleolar phosphoprotein B23), a nuclear shuttling chaperone protein that, among other things, functions in ribosome biogenesis. The nucloeolar localization motif present within cardioviral 2A and the consistent colocalization of 2A and nucleophosmin has led to the hypothesis that nucleophosmin associates with 2A and aids in the nucleolar localization of this picornavirus protein. This would allow cardioviral 2A to enter the nucleus camouflaged as a ribosomal protein, where Pol I transcribes rRNA genes and ribosomal subunit assembly occurs (Aminev et al., 2003a). 2A may target the nucleolus to alter ribosomal biogenesis in some way, promoting viral IRES-dependent translation and/or 2A inclusion within ribosomes (Medvedkina et al., 1974). The EMCV 3BCD minor precursor protein has also been shown to associate with nucleophosmin and enter the nucleolar compartment via an NLS present in the 3D amino acid sequence, independent of infection (Aminev et al., 2003b). In agreement with studies of HRV 16, this NLS is located at the very N-terminus of 3D and mimics a yeast ribosomal protein NLS rather than the basic NLS suggested for poliovirus.

### FMDV has unique interactions with the nucleus

**Figure 6:**
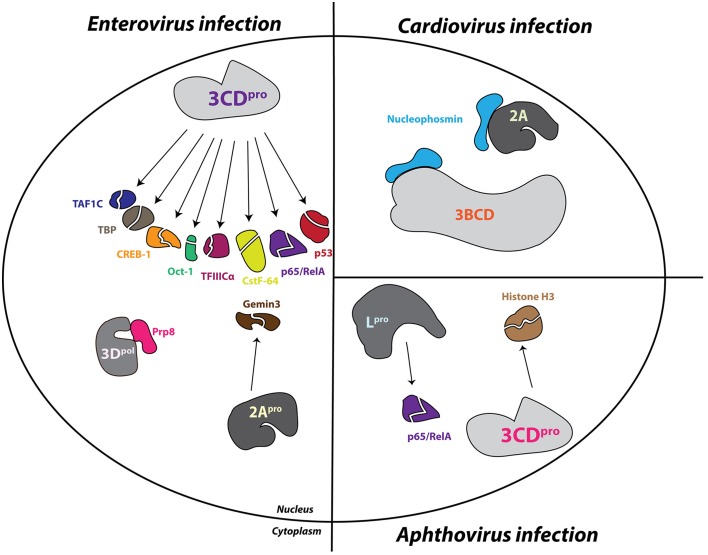
**Picornavirus proteins enter the nucleus and alter nuclear-resident proteins**. During enterovirus infections, the viral proteinase 3CD/3C (3CD^pro^) enters the nucleus and degrades TATA box-binding protein-associated factor RNA polymerase I subunit C (TAF 1C), leading to inhibition of RNA Polymerase I (Pol I) transcription. Pol III-driven transcription is also inhibited by 3CD^pro^, through cleavage of general transcription factor IIIC polypeptide 1 (TFIIICα). Pol II transcription, including cellular mRNA production, is terminated in enterovirus infected cells through cleavage of TATA-box-binding-protein 1 (TBP1), cyclic AMP-responsive element-binding protein 1 (CREB-1), and POU domain, class 2, transcription factor 1 (Oct-1) by 3CD^pro^. The transcription factors cellular tumor antigen p53 (p53), cleavage stimulation factor subunit 2 (CstF-64), and the NF-κB subunit p65/RelA (p65/RelA) are also degraded in a 3CD^pro^ dependent manner during enterovirus infections. Furthermore, the enteroviral polymerase 3D (3D^pol^) associates with the splicing factor pre-mRNA processing factor 8 (Prp8), causing dysregulation of splicing, and the 2A proteinase (2A^pro^) cleaves Probable ATP-dependent RNA helicase DDX20 (Gemin3). Cardiovirus infection causes the nuclear localization of both 2A and precursor protein 3BCD, both of which may associate with nucleophosmin. Infection with FMDV causes the cleavage of both Histone H3 as well as p65/RelA within the nucleus, following the entry of both L proteinase (L^pro^) and 3CD^pro^.

FMDV has distinct interactions with the transcription apparatus of cells. In contrast to other picornaviruses, FMDV modulates cellular transcription by targeting the regulation of transcriptionally active chromatin through the cleavage of the nucleosome component histone H3 (Griger and Tisminetzky, 1984). The FMDV 3C proteinase is capable of histone H3 cleavage *in vitro*, thus inhibiting euchromatin formation and subsequent transcription (Falk et al., 1990; Tesar and Marquardt, 1990). Transient transfection of FMDV 3ABC precursor protein expression constructs causes histone H3 degradation; however, a recent report suggests that the NLS motif MRKTKLAPT present in FMDV 3D is responsible for transporting 3CD to the nucleus, as well as GFP fused to this NLS, in the absence of infection (Capozzo et al., 2002; Sanchez-Aparicio et al., 2013). It is possible that the cleavage observed upon transfection of FMDV 3ABC was attributable to infection with vaccinia virus encoding a T7 RNA polymerase prior to transfection to facilitate 3ABC expression from the T7 promoter-containing plasmid. Nonetheless, it appears that similar to the enteroviruses, FMDV 3CD enters the nucleus via an NLS present in the 3D amino acid sequence, allowing proteinase functions to target cellular proteins within the nucleus. The L proteinase of FMDV has also been shown to localize to the nucleus of porcine cells during wild type FMDV infection (De Los Santos et al., 2007). As noted above, FMDV L entry into the nucleus correlates with a decrease in the transcription of antiviral IFN-β mRNA (De Los Santos et al., 2006). The resulting decrease in IFN production is not a result of FMDV infection causing inhibition of NF-κB translocation to the nucleus, reductions in NF-κB mRNA transcription, or a result of widespread degradation of nuclear proteins, but instead is a consequence of decreases in the nuclear levels of the p65/RelA protein subunit of NF-κB, as has also been observed during enterovirus infection. The L proteinase appears to be necessary and sufficient for the degradation of p65/RelA, as infection with the murine-specific TMEV encoding FMDV L also leads to FMDV L nuclear localization and disappearance of p65/RelA in the nucleus of infected cells (De Los Santos et al., 2007). Furthermore, L proteinase catalytic activity is required for p65/RelA degradation. The motif present within FMDV L responsible for imparting nuclear localization was mapped to a SAF-A/B, Acinus, and PIAS (SAP) domain within L, a protein domain that is associated with nuclear retention of some proteins involved in transcriptional control. Virus containing a double mutation in the SAP region of L showed altered nuclear localization upon infection (De Los Santos et al., 2009) (Figure 6).

The inhibition of cellular gene expression by picornaviruses is carried out in diverse ways that target translation as well as splicing and transcription. To affect the latter two processes, some picornavirus proteins enter the nucleus of the infected cell. The pro-viral effects of reduced cellular gene expression are two-fold: to liberate cellular proteins with functions advantageous to viral replication and to re-route metabolic energy from cell-specific to viral-centric functions. It is somewhat surprising that the RNA-dependent RNA polymerase, 3D, of many picornaviruses becomes localized to the nucleus of infected cells, because it would seem that maximizing viral RNA production in the cytoplasm would be prioritized during the infectious cycle. The fact that many picornavirus 3D proteins contain an NLS suggests that there are specific functions of 3D and/or the precursor 3CD in the nucleus, some of which remain to be uncovered, which are balanced with the polymerase function of 3D in the cytoplasm. It is a given that further experimentation will continue to reveal the elegant ways in which picornaviruses alter the cellular functions that occur in the nucleus and the way in which viral proteins are able to infiltrate the command center of the cell to appropriate cellular components and functions for their benefit.

## Conclusions

Picornaviruses have traditionally been labeled as “cytoplasmic” RNA viruses as a result of the subcellular region in which these viruses produce viral proteins and replicate their genomic RNA molecules. Indeed, picornaviruses are able to complete the replicative cycle and produce infectious progeny in nucleus-free cytoplasts and cytoplasmic extracts (Pollack and Goldman, 1973; Follett et al., 1975; Molla et al., 1991; Barton and Flanegan, 1993; Svitkin and Sonenberg, 2003). However, it is not clear that enucleation treatments, e.g., with cytochalasin B, do not disrupt the nuclear envelope and allow the escape of nuclear-resident proteins or that cytoplasmic extracts do not contain significant concentrations of nuclear-shuttling or nuclear-resident proteins as a result of nuclear envelope leakage during cellular fractionation. It is significant that following cytochalasin B enucleation treatment, poliovirus capsid synthesis and virus growth are less efficient compared to infection of nucleated, untreated cells with a final yield from enucleated cells one-fifth of that from nucleated cells (Pollack and Goldman, 1973). Some studies have even provided evidence of a nuclear requirement of poliovirus early in infection, as virion RNA is able to replicate in the absence of a cell nucleus but transfection of replicative form RNA into enucleated cells produced no detectable viral progeny (Detjen et al., 1978). As discussed in this review, it is clear that picornaviruses make extensive use of nuclear-resident and nuclear-shuttling proteins to promote viral replication. The nuclear shuttling proteins PCBP2 and PTBP1 are particularly important for enterovirus translation as well as mediating the template usage switch required for RNA replication to proceed. Furthermore, PCBP2 and PABP1 may function in the circularization of genomic RNA templates to facilitate negative-sense intermediate RNA production. Additionally, the nuclear-resident hnRNP C1/C2 is necessary for the efficient synthesis of poliovirus genomic RNA molecules from negative-sense intermediate RNA forms. Following the early rounds of translation in which the limited quantities of nuclear proteins in the cytoplasm are sufficient for viral protein production, picornavirus proteins 3CD/3C, 2A, and L induce alterations to the NPC. Cleavage of nucleoporins by enteroviral 2A and 3CD/3C or hyperphosphorylation of nucleoporins triggered by cardioviral L results in dysregulation of nucleocytoplasmic trafficking and subsequent loss of nuclear compartmentalization of particular proteins within picornavirus infected cells. The presence of these nuclear proteins with functions critical to viral replication (including RNA-binding capabilities) allows for the amplification of viral progeny. Also as a result of deviations from standard nucleocytoplasmic trafficking, picornaviruses are able to disrupt the proper signaling pathways that allow for a strong innate immune response, including interferon production. Finally, picornavirus proteins enter the nucleus of infected cells to carry out functions related to host-cell transcription inhibition, such as degradation of transcription factors, allowing metabolic energy and proteins sequestered in cellular roles to be redirected to virus-centric demands. Picornaviruses display a prominent level of direct and indirect interactions with the nucleus, interactions that have the potential to reveal pathogenic mechanisms and unique antiviral strategies as well as insights into cell biology through more detailed study. The fact that viruses of the *Picornaviridae* family have evolved to orchestrate a well-balanced promotion of viral replication with host-cell attenuation of nucleocytoplasmic trafficking demonstrates that interactions with the nucleus are functional and that these coding-capacity-limited cytoplasmic RNA viruses are master manipulators of even the most complex of cellular processes.

### Conflict of interest statement

The authors declare that the research was conducted in the absence of any commercial or financial relationships that could be construed as a potential conflict of interest.
